# Study on the Effects of Polyphenols on the Properties, Microstructure, and Digestibility of Rice Protein Gel and the Interaction Mechanisms Between Polyphenols and Rice Protein

**DOI:** 10.3390/foods15111854

**Published:** 2026-05-24

**Authors:** Anna Wang, Mengran Fan, Ligen Wu

**Affiliations:** 1 College of Food Science and Engineering, Henan University of Technology, Zhengzhou 450002, China; wanganna2017@126.com (A.W.); mengran787@163.com (M.F.); 2 National Engineering Research Center of Wheat and Corn Further Processing, Henan University of Technology, Zhengzhou 450002, China

**Keywords:** rice protein gel, polyphenol–protein interaction, antioxidant capacity, molecular docking, in vitro digestibility

## Abstract

Rice protein has limited gelation properties, restricting its food applications. This study added four polyphenols—catechin (C), epicatechin (EC), tannic acid (TA), and proanthocyanidins (PC)—to rice protein to investigate their effects on gel rheology, in vitro digestibility, and microstructure. Multi-spectroscopy and molecular docking were used to explore interaction mechanisms. During the temperature sweep (95 °C), PC- and TA-composite gels (GRP-PC, GRP-TA) showed storage moduli slightly higher than the pure rice protein gel (GRP), while GRP-C and GRP-EC (C- and EC-composite gels) were similar to GRP. In frequency sweep (25 °C), GRP had the highest modulus, followed by GRP-PC > GRP-TA > GRP-EC > GRP-C. Polyphenols reduced total digestibility (from 77.4% to 67.6–75.2%). All polyphenol-complexed gels showed markedly improved ABTS (2,2′-azino-bis(3-ethylbenzothiazoline-6-sulfonic acid)) and DPPH (2,2-diphenyl-1-picrylhydrazyl) radical scavenging activities. C and EC induced loosely crosslinked microstructures, whereas TA and PC formed sheet-like aggregates. Fluorescence quenching was predominantly static, with quenching rates TA > PC > EC > C. Binding constants followed the same order. Thermodynamic parameters (Δ*H* > 0, Δ*S* > 0, Δ*G* < 0) indicated hydrophobic interactions as the driving force. Molecular docking revealed that PC formed the most hydrogen bonds (8) with rice glutelin, followed by TA (4), C (5), and EC (3). These findings provide data support for designing rice protein-based functional foods.

## 1. Introduction

Rice protein (RP) has garnered certain attention in the food industry due to its high content of essential amino acids [1], low allergenicity [2], absence of pigment interference and anti-nutritional factors [3], as well as its mild flavor [4]. Previous studies have indicated that rice protein may possess health benefits such as antidiabetic, cholesterol-lowering, and anticancer effects [5]. With the development of plant-based foods, the market demand for rice protein has gradually increased, and its application fields are expanding [6]. Although rice protein possesses techno-functional properties such as gelation, emulsi-fication, and oil-binding capacity, which are important for food applications, pure rice protein gels have certain limitations in functional properties [7]. Therefore, investigating the interactions between rice protein and other food components, such as polyphenols, holds promise for improving the functional properties of the gels and providing a basis for the development of foods with enhanced nutritional value [8].

Polyphenols are natural compounds containing multiple phenolic hydroxyl groups, widely present in plants, and exhibit various biological activities, including antioxidant, anti-inflammatory, immunomodulatory, and cardiovascular protective effects [9,10]. The physicochemical properties of polyphenols vary depending on their structural differences. For example, catechin (C) and epicatechin (EC) are flavanol-type polyphenols with relatively low molecular weights and fewer phenolic hydroxyl groups [11]. Tannic acid (TA) belongs to the hydrolyzable tannin class, has a larger molecular weight, and contains multiple phenolic hydroxyl groups [12]. Proanthocyanidins (PC), another type of condensed tannins, are polymerized from flavanol units and are characterized by a high molecular weight and abundant phenolic hydroxyl groups [13]. These structural differences may influence the manner and extent of their interactions with proteins, thereby exerting different regulatory effects on the properties of rice protein gels.

Previous studies have indicated that polyphenols can form complexes with proteins through covalent or non-covalent interactions [14]. Proteins can serve as carriers to improve the stability of polyphenols, while the introduction of polyphenols may also alter the solubility, emulsifying properties, and gel-forming ability of proteins [15]. In gel systems, the addition of polyphenols may influence protein aggregation behavior, network structure, as well as subsequent digestibility and antioxidant activity [16]. However, comparative studies on the effects of polyphenols with different structures on rice protein gelation, digestibility, and mechanical behavior are still relatively limited.

To this end, four representative polyphenols with distinct structures—catechin, epicatechin, tannic acid, and proanthocyanidins—were selected and individually compounded with rice protein to prepare composite gels for multi-level comparative analysis. The study first examined the effects of polyphenols on the mechanical properties of the gels via rheological analysis, followed by evaluation of changes in protein digestibility and radical scavenging capacity during in vitro simulated digestion. Scanning electron microscopy was used to observe the microstructural characteristics of the gels, while UV-Vis spectroscopy, fluorescence spectroscopy, and molecular docking simulations were employed to explore the interaction modes between polyphenols and rice protein. By establishing the correlations among ‘macroscopic properties—functional performance—microstructure—molecular mechanisms’, this study provides a theoretical basis for the targeted design of rice protein-based foods with specific digestive behaviors and antioxidant functions [17].

## 2. Materials and Methods

### 2.1. Standards and Reagents

The main chemical reagents used in this study were as follows.

Rice protein (purity ≥ 90%): Shaanxi Shenghe Jianyuan (Xi’an, China).

Catechin (95%), epicatechin (95%), proanthocyanidins (99%), tannic acid (95%), trichloroacetic acid (AR), 2,2′-azino-bis(3-ethylbenzothiazoline-6-sulfonic acid) (ABTS, BR), 2,2-diphenyl-1-picrylhydrazyl (DPPH), α-amylase (50 U·mg^−1^), pepsin (3000 U·mg^−1^), porcine bile salt (AR): Shanghai Macklin Biochemical Technology Co., Ltd. (Shanghai, China).

Pancreatin (4000 U·mg^−1^): Shanghai Yuanye Bio-Technology Co., Ltd. (Shanghai, China).

30% hydrogen peroxide (AR), potassium chloride (AR), sodium bisulfite (AR), potassium dihydrogen phosphate (AR), sodium bicarbonate (AR), sodium chloride (AR), magnesium chloride (AR), ammonium chloride (AR): Tianjin Kemiou Chemical Reagent Co., Ltd. (Tianjin, China).

Hydrochloric acid (AR): Xilong Scientific Co., Ltd. (Shantou, Guangdong, China).

Bicinchoninic acid (BCA) protein assay kit: Beijing Solarbio Science & Technology Co., Ltd. (Beijing, China).

### 2.2. Instruments and Equipment

The main instruments and equipment used in this study include the DF-101S constant-temperature heating magnetic stirrer (Henan Baize Instrument Co., Ltd., Zhengzhou, China); the field emission scanning electron microscope (FEI Company, Hillsboro, OR, USA; ZEISS, Oberkochen, Germany); the vacuum freeze dryer (Beijing Boyikang Experimental Instrument Co., Ltd., Beijing, China); the F98 fluorescence spectrophotometer (Shanghai Lengguang Technology Co., Ltd., Shanghai, China); the UV7600 double-beam ultraviolet-visible spectrophotometer (Shanghai Lengguang Technology Co., Ltd., Shanghai, China); the TA.XT plus Texture Analyzer (Stable Micro Systems Ltd., Surrey, UK).

### 2.3. Sample Preparation

#### 2.3.1. Preparation of Gel Samples

A 24% (*w*/*w*) rice protein (RP) solution (total mass 4.5 g) was prepared using Tris-HCl buffer (pH 9.0) containing 0.0097 M CaCl_2_ as the solvent. To this solution, 0.080 g of C, 0.080 g of EC, 0.234 g of TA, and 0.045 g of PC were separately added, mixed thoroughly, and then subjected to ultrasonication at 400 W for 70 min. After ultrasonication, the solution was heated at 65 °C for 15 min, followed by heating to 95 °C and held for 96.4 min. Upon completion of heating, the samples were cooled with ice water and stored at 4 °C for 24 h to obtain protein gels. The rice protein gel and the composite gels of rice protein with catechin, epicatechin, tannic acid, and proanthocyanidins were designated as GRP, GRP-C, GRP-EC, GRP-TA, and GRP-PC, respectively. The heating time of 96.4 min was determined from a response surface optimization of pure rice protein gel and was kept constant for all samples to allow direct comparison. The amounts of catechin (0.080 g), epicatechin (0.080 g), tannic acid (0.234 g), and procyanidin B2 (0.045 g) were determined by preliminary experiments as the minimum effective dosage for each polyphenol to form a stable, self-standing composite gel.

#### 2.3.2. Preparation of Solution Samples

Rice protein was dissolved in deionized water at a concentration of 10 mg·mL^−1^, stirred for 1 h, and then centrifuged to obtain the supernatant. Solutions of C, EC, and TA were prepared in sodium carbonate–sodium bicarbonate buffer (pH 9.0) at a concentration of 1.18 mmol L^−1^; PC was prepared in the same buffer at a concentration of 0.01 mol·L^−1^. Aliquots of 2 mL of the RP supernatant were mixed with different volumes of each polyphenol solution as follows: C at 5, 10, 20, 35, and 50 μL; EC at 5, 10, 15, 25, 35, and 50 μL; TA at 5, 10, 20, 35, and 50 μL; and PC at 5, 10, 20, 30, 40, and 50 μL. After mixing, portions of the mixtures were heated at 310 K for 2 h to obtain polyphenol–rice protein complex solutions at 298 K and 310 K. The complexes of RP with C, EC, TA, and PC were designated as RP-C, RP-EC, RP-TA, and RP-PC, respectively. The volume series for each polyphenol was designed to cover comparable concentration ranges for fluorescence quenching analysis. Slight variations in the number of points (e.g., five for C, six for EC) reflect minor preliminary adjustments to optimize curve fitting; all series yielded good linearity.

### 2.4. Dynamic Rheological Properties

#### 2.4.1. Temperature Sweep

A fixed strain of 0.1% and a fixed frequency of 1.0 Hz were applied. The sample was equilibrated at 25 °C for 60 s and then heated linearly from 25 °C to 95 °C at a rate of 2 °C·min^−1^. The measurement gap was set to 1 mm [18].

#### 2.4.2. Frequency Sweep

Dynamic rheological analysis was performed using a rheometer. First, the linear viscoelastic region of all samples was determined by amplitude sweep (strain range 0.01–100%, fixed frequency 1 Hz). To ensure that the gel network structure was not disrupted during the test, subsequent frequency sweeps were conducted within the determined linear viscoelastic region using a fixed strain value of 0.1%, over a frequency range of 0.1–16 Hz. The test temperature was maintained at 25 °C, and the plate gap was set to 1 mm [19].

### 2.5. Texture Profile Analysis (TPA)

TPA was performed using a TA.XT plus Texture Analyzer equipped with a P/0.5 cylindrical probe (diameter 12.7 mm). The gel samples were prepared as described in Section 2.3.1. The gel was tested after being removed from the refrigerator and equilibrated at room temperature for 1 h. The test parameters were set as follows: pre-test speed 1.0 mm·s^−1^, test speed 1.0 mm·s^−1^, post-test speed 1.0 mm·s^−1^, compression distance 10 mm, trigger force 5 g, and time interval between two compressions 3 s [20].

### 2.6. Simulated In Vitro Digestion

In vitro simulated digestion was performed according to the INFOGEST 2.0 static digestion method [21]. Simulated salivary fluid (SSF), simulated gastric fluid (SGF), and simulated intestinal fluid (SIF) were prepared. Gel samples (0.5 g each) were mixed with 7.5 mL of salivary electrolyte solution and 2.5 mL of α-amylase solution (enzyme activity 75 U·mL^−1^), and then incubated in a water bath at 37 °C and 115 rpm for 10 min.

After the oral digestion step, the pH of the solution was adjusted to 2.0 using HCl solution. Then, 4 mL of pepsin solution (2000 U·mL^−1^), 16 mL of gastric electrolyte solution, and 10 μL of 0.3 mol L^−1^ CaCl_2_ were added. The mixture was incubated at 37 °C with constant shaking at 115 rpm. At 0, 30, 60, 90, and 120 min, two 1 mL aliquots were taken, and the enzyme was inactivated by heating in a boiling water bath. After cooling, the pH was adjusted to 7.0 using 0.1 mol·L^−1^ NaOH solution. The samples were then centrifuged, and the supernatants were collected for subsequent analysis.

After 120 min of gastric digestion, the pH of the sample was adjusted to 7.0. A pancreatin-bile salt mixture (8 mg·mL^−1^ pancreatin and 50 mg·mL^−1^ bile salts) was then added at a volume fraction of 0.2% of the total volume. The mixture was incubated at 37 °C with constant shaking. At 0, 30, 60, 90, and 120 min, two 1 mL aliquots were taken, and the enzyme was inactivated by heating in a boiling water bath for 2 min. After cooling, the pH was adjusted to 7.0 using NaOH solution. The samples were then centrifuged, and the supernatants were collected for subsequent analysis.

### 2.7. Determination of Protein Digestibility

Equal volumes of 20% trichloroacetic acid were used to precipitate the test samples before and after digestion. After standing for 30 min, the samples were centrifuged at 8000 rpm for 10 min. The supernatant was discarded, and the precipitate was dissolved in 1 M NaOH. The protein content in the precipitate was determined using a BCA protein assay kit. The absorbance of the undigested sample (0 min) was recorded as *A*_1_, and that of the digested sample as *A*_0_. The protein digestibility was calculated according to the following formula:

(1) $$\mathrm{Digestibility}\;(\%)\;=\;\frac{A_{1}-A_{0}}{A_{1}}\times 100$$

### 2.8. Determination of ABTS^+^ Radical Scavenging Capacity

ABTS and potassium persulfate were separately dissolved in distilled water to prepare stock solutions of 7.0 mmol·L^−1^ and 5.0 mmol·L^−1^, respectively [22]. Equal volumes of the two stock solutions were mixed to obtain an ABTS-potassium persulfate mixture (final concentrations: ABTS 3.5 mmol·L^−1^, potassium persulfate 2.5 mmol·L^−1^), which was then stored in the dark for 12 h. Prior to use, the mixture was diluted with PBS (pH 7.4) to an absorbance of 0.70–0.75 at 734 nm, which served as the ABTS working solution. A 10 μL sample was mixed with 250 μL of the working solution and allowed to react in the dark for 30 min. The absorbance was measured at 734 nm (*A*_1_). A blank (using absolute ethanol instead of the sample, *A*_0_) and a control (using absolute ethanol instead of ABTS, *A*_2_) were also prepared. The scavenging rate was calculated according to the following formula:

(2) $$\mathrm{Scavenging}\;\mathrm{rate}\;(\%)\;=\;\frac{{1-(A}_{1}-A_{2})}{A_{0}}\times 100$$

### 2.9. Determination of DPPH· Radical Scavenging Rate

The DPPH· radical scavenging rate was calculated by comparing the change in absorbance of the sample before and after the reaction. The specific procedure was as follows: DPPH· was dissolved in absolute ethanol to prepare a working solution at a concentration of 40 mg·mL^−1^. A 10 μL sample was mixed with 250 μL of the DPPH· solution and allowed to react in the dark for 30 min, after which the absorbance was measured at 517 nm (A_1_) [23]. A blank control (A_0_) and a background control (A_2_) were also set up in the same manner as for the ABTS assay. The scavenging rate was calculated using the same formula as described for ABTS^+^ (Equation (2)).

### 2.10. Scanning Electron Microscopy

The gel samples were freeze-dried and naturally broken to expose the cross-sectional area. The samples were attached to aluminum sample holders using double-sided conductive tape and then sputter-coated with gold to render them conductive. Scanning was performed at magnifications of 400×, 1000×, and 1120× to obtain SEM images.

### 2.11. UV-Vis Spectroscopy Method

Using a UV7600 double-beam UV-Vis spectrophotometer and referring to the experimental method of Hu, X. et al. [24], a 0.1 mol L^−1^ sodium carbonate-sodium bicarbonate solution (pH 9.0) was used as a reference. The UV-Vis absorption spectra of the complex solutions were recorded in the range of 200 to 450 nm.

### 2.12. Fluorescence Spectroscopy Method

Using an F98 fluorescence spectrophotometer and referring to the method of Duan, S.T. [25] with slight modifications, the excitation wavelength was set to 280 nm, and the fluorescence intensity of the complex solution was measured over an emission wavelength range of 300 to 450 nm.

### 2.13. Synchronous Fluorescence Spectroscopy

Synchronous fluorescence spectra were recorded with Δλ = 15 nm and Δλ = 60 nm. The excitation wavelength was scanned from 250 to 500 nm, and the fluorescence intensity of the complex solution was measured.

### 2.14. Three-Dimensional Fluorescence Spectroscopy

The fluorescence intensity of the complex solution was measured over excitation and emission wavelength ranges of 250 to 500 nm.

### 2.15. Molecular Docking Simulation of Polyphenols with Rice Protein

The rice protein sequence (UniProt ID: Q10AZ7) was retrieved from the UniProt database (https://www.uniprot.org/, accessed on 1 March 2026). The three-dimensional structures of the four polyphenols, namely proanthocyanidins (CID: 108065), epicatechin (CID: 9064), catechin (CID: 107905), and tannic acid (CID: 16129778), were downloaded from the PubChem chemical database (https://pubchem.ncbi.nlm.nih.gov/, accessed on 1 March 2026) in SDF format. The SDF files were converted to PDB format using the Open Babel tool-3.1.1. AutoDockTools-v4.2.6 was used to add hydrogen atoms to the protein, compute Gasteiger charges, and set it as the receptor. Meanwhile, the polyphenol ligands were processed to detect rotatable bonds and saved in PDBQT format. During the docking procedure, a grid box was set to cover the entire protein active pocket, and 50 independent docking calculations were performed using the Lamarckian genetic algorithm, with all other parameters set to default values. The best docking conformation was selected based on the lowest binding energy principle. Finally, Chimera-1.1.9 was used for three-dimensional visualization of the molecular docking details, displaying the overall binding mode and local interacting residues, and Discovery Studio Visualizer-2025 Client was employed to generate two-dimensional interaction diagrams to analyze key interactions such as hydrogen bonds and hydrophobic interactions.

### 2.16. Statistical Analysis

All experiments were performed in at least three independent replicates (n ≥ 3). Statistical analysis was conducted using one-way analysis of variance (ANOVA) followed by Tukey’s post hoc test. Differences were considered significant at *p* < 0.05. Different lowercase letters in figures indicate significant differences among samples.

### 2.17. FTIR Measurement 

Freeze-dried polyphenol-protein gel powder was mixed with KBr at a ratio of 1:200 and ground together, then pressed into translucent pellets. FTIR spectra were recorded in transmission mode using a Nicolet FTIR spectrometer (Thermo Fisher Scientific, Madison, WI, USA) in the range of 4000–400 cm^−1^ with a resolution of 4 cm^−1^ and 64 scans per sample. A background spectrum (air) was collected before each sample and automatically subtracted. All measurements were performed at room temperature (25 °C). The raw spectra were baseline corrected and deconvoluted using OMNIC software. Second derivative and Fourier self deconvolution were applied to resolve the overlapped amide I band (1600–1700 cm^−1^). Curve fitting was performed with a Gaussian function using Peakfit v4.12 software. The fitted sub peaks were assigned to secondary structures according to literature [26] (α helix ~1650 cm^−1^, β sheet ~1630 and ~1690 cm^−1^, β turn ~1670 cm^−1^, random coil ~1640 cm^−1^). The relative contents of protein secondary structures were calculated from the integrated areas of the fitted sub peaks.

## 3. Results and Analysis

### 3.1. Effect of Polyphenols on the Rheological Properties of Rice Protein Gels

#### 3.1.1. Temperature Sweep Analysis

**Figure 1:**
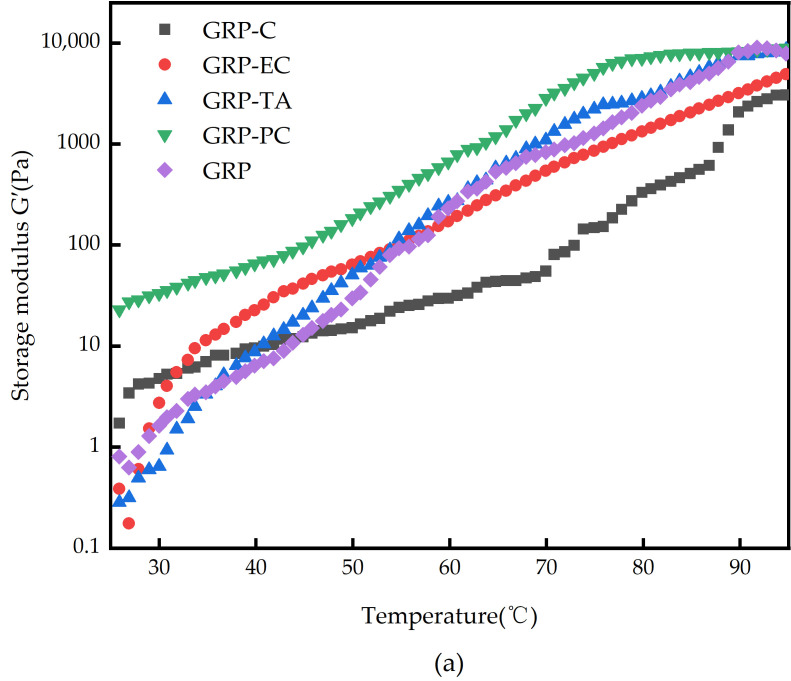

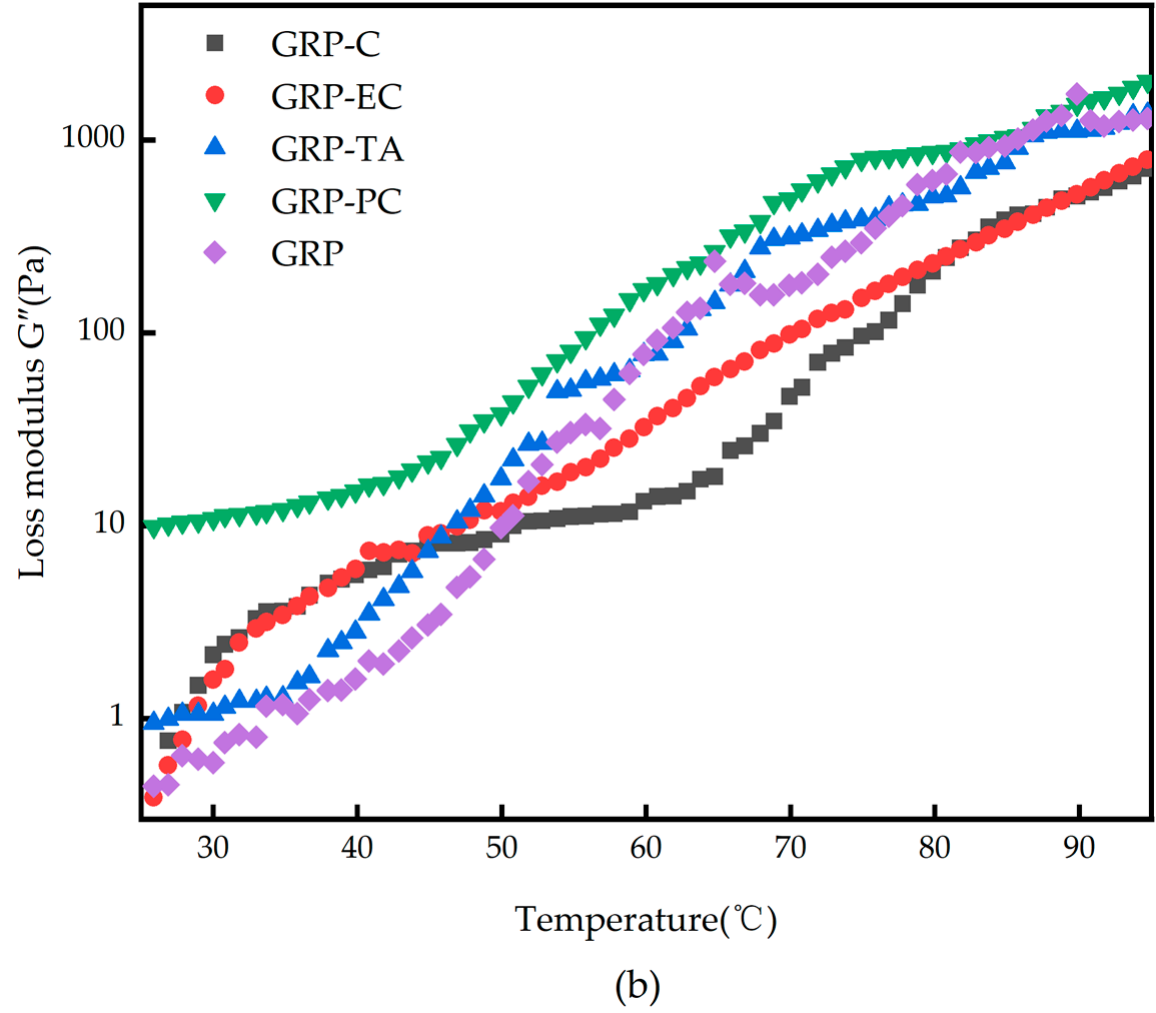
Temperature sweep (25–95 °C) of rice protein gels with and without polyphenols. (**a**) Storage modulus (G′), (**b**) Loss modulus (G″). Note: GRP: rice protein gel; GRP-C, GRP-EC, GRP-TA, and GRP-PC: the composite gels of rice protein with catechin, epicatechin, tannic acid, and proanthocyanidins, respectively.

The aggregation behavior and network structure of proteins are key factors affecting their rheological properties [27]. Figure 1 shows the changes in storage modulus (G′) and loss modulus (G″) of the protein gel (GRP) and the four polyphenol–protein composite gels during heating from 25 °C to 95 °C. At the initial stage of heating (25–60 °C), all samples exhibited low moduli, with G″ close to or slightly higher than G′, indicating that the systems were predominantly viscous. Above 60 °C, G′ increased rapidly and exceeded G″, suggesting protein denaturation and aggregation, and the gel network began to form.

At the final heating temperature (95 °C), the G′ values of all samples were higher than their G″ values across the entire frequency range, indicating typical elastic gel behavior. At 95 °C, the final G′ values of GRP-PC, GRP-TA, and GRP were comparably high and nearly superimposable. GRP-EC and GRP-C exhibited distinctly lower G′ values, with GRP-C being the lowest. This result may be related to the effect of polyphenols on the thermal stability of the protein network, as the addition of polyphenols altered the thermal aggregation behavior of the protein [28]. The higher G′ values of GRP-PC and GRP-TA at 95 °C suggest that these polyphenols promote more extensive protein cross-linking during heating, likely due to their higher molecular weight and greater number of phenolic hydroxyl groups. This results in a gel network with higher cross-linking density upon cooling.

#### 3.1.2. Frequency Sweep Analysis

**Figure 2:**
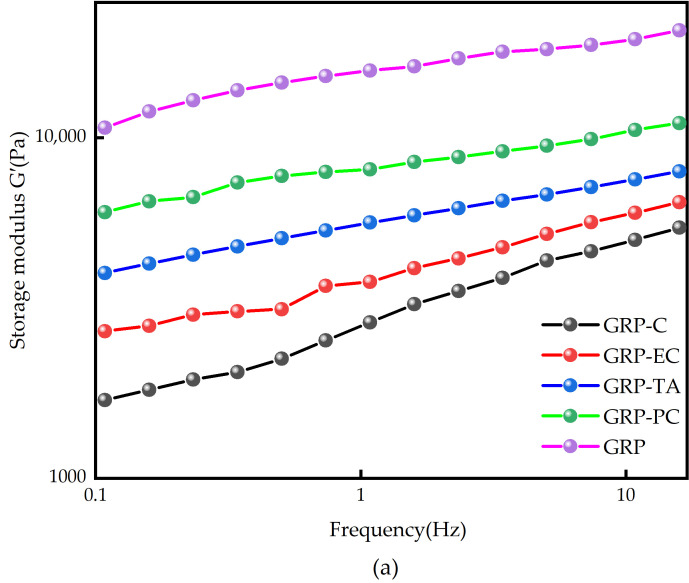

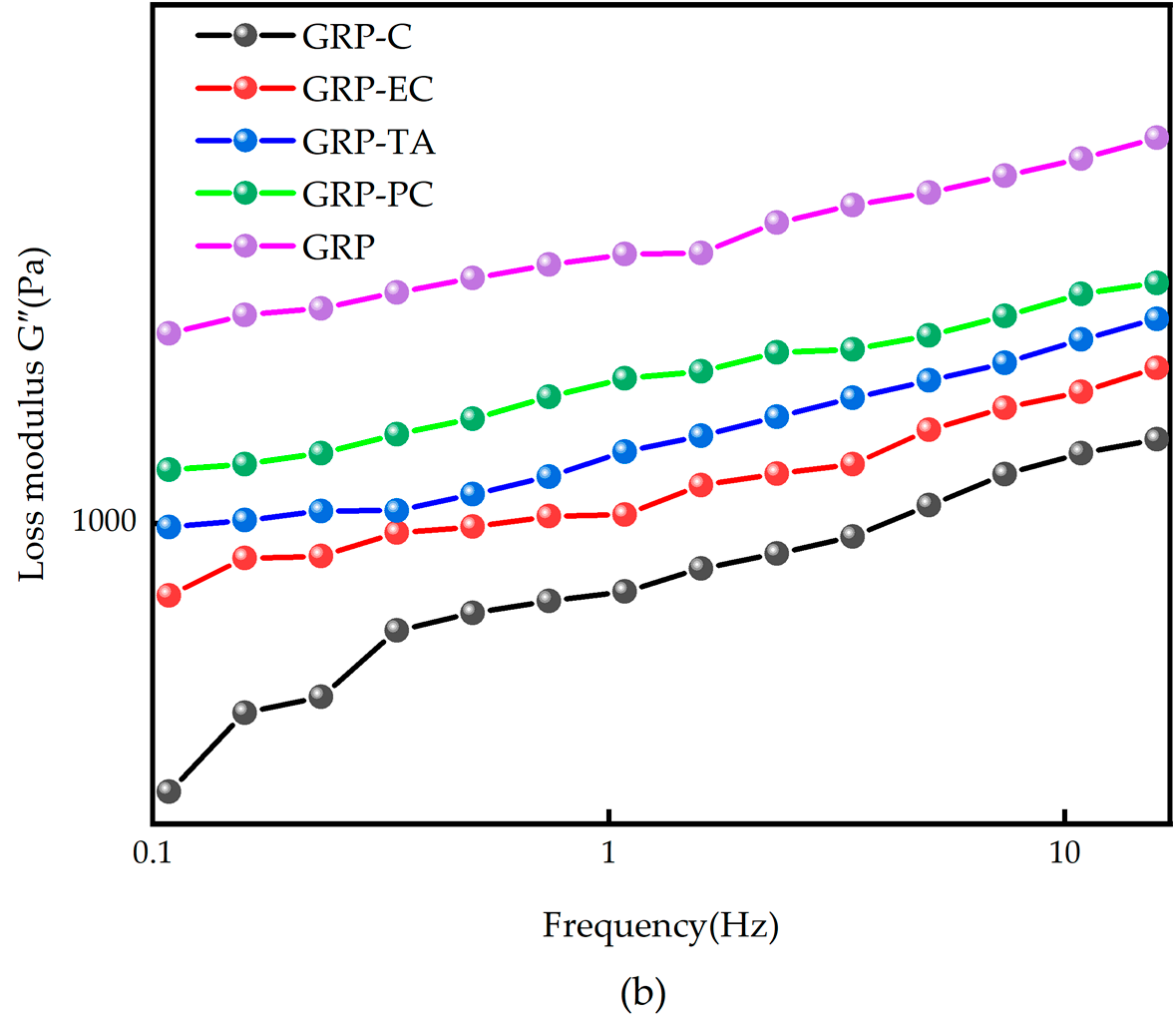
Frequency sweep (0.1–20 Hz, 25 °C) of rice protein gels. (**a**) Storage modulus (G′), (**b**) Loss modulus (G″). Note: GRP: rice protein gel; GRP-C, GRP-EC, GRP-TA, and GRP-PC: the composite gels of rice protein with catechin, epicatechin, tannic acid, and proanthocyanidins, respectively.

Figure 2 presents the frequency sweep results (0.1–16 Hz) for each sample at 25 °C. For all samples, G′ remained greater than G″ across the entire frequency range without crossover, indicating that the gel networks remained intact under dynamic shear, consistent with typical physical gel characteristics. The G′ of GRP increased from approximately 10,700 Pa to 20,670 Pa with increasing frequency, which was significantly higher than that of the polyphenol-composite gels. The G′ values of the polyphenol-composite gels followed the order: GRP-PC > GRP-TA > GRP-EC > GRP-C. At 16 Hz, GRP-PC exhibited a G′ of approximately 11,043 Pa, GRP-TA approximately 7964 Pa, GRP-EC approximately 6463 Pa, and GRP-C approximately 5444 Pa, while the G′ of GRP was approximately twice that of GRP-PC.

The storage modulus of GRP in the frequency scan (20,670 Pa) was significantly higher than that of all polyphenol–composite gels, which differs from the results of the temperature scan. This may be attributed to the fact that after 24 h of refrigeration at 4 °C, the protein molecules in the GRP network underwent sufficient rearrangement, forming a dense network. In contrast, the binding of polyphenols to proteins can induce changes in protein secondary structure. This is supported by our FTIR analysis (Supplementary Figure S1), which measured the secondary structure changes in the gels, including a decrease in β-sheet content and an increase in random coil content [29]. This may be one of the intrinsic reasons for the observed differences in gel network.

However, the distinct behavior of TA/PC versus C/EC suggests an additional factor: the strong crosslinks formed by TA and PC, while beneficial for gelation during heating, restrict network rearrangement during cold storage, resulting in a rigid but shear-sensitive structure [30]. In contrast, the weak interactions of C and EC do not hinder cold-storage rearrangement, but they also fail to reinforce the network, leading to consistently low G′.

The temperature sweep and frequency sweep results collectively indicate that the addition of all four polyphenols altered the rheological behavior of rice protein gels. During the gelation process, the final moduli of the polyphenol–composite gels were comparable to, or even higher than, those of the pure protein gel. However, under dynamic oscillatory conditions, their moduli were significantly lower than that of the pure protein gel, and the extent of the effect correlated with binding ability. PC and TA preserved gel viscoelasticity better than EC and C. These differences in rheological properties provide a basis for further understanding the effects of polyphenols on the digestibility and microstructure of protein gels.

### 3.2. Effect of Polyphenols on TPA of Rice Protein Gels

Texture profile analysis was performed to quantify the large-deformation mechanical properties of the gels (Table 1). The hardness of GRP (239.73 ± 5.94 g) was the highest among the four formulations. GRP-C (233.65 ± 3.71 g) and GRP-EC (210.01 ± 3.31 g) showed comparably high hardness, while GRP-TA exhibited significantly lower hardness (135.98 ± 13.72 g, *p* < 0.05). GRP-PC displayed an intermediate hardness (159.75 ± 3.21 g), which was significantly higher than GRP-TA (*p* < 0.05) but lower than GRP (*p* < 0.05). Springiness, cohesiveness, gumminess, and chewiness followed the same order: GRP ≈ GRP-C ≈ GRP-EC > GRP-PC > GRP-TA. Springiness, cohesiveness, gumminess, and chewiness followed the same order: GRP ≈ GRP-C ≈ GRP-EC > GRP-TA (Table 1).

Importantly, the TPA hardness order (GRP ≈ GRP-C > GRP-EC > GRP-PC > GRP-TA) did not align with the small-deformation storage modulus order from the frequency sweep (GRP >> GRP-PC > GRP-TA > GRP-EC > GRP-C). GRP-TA, which exhibited a moderately high G′ (7964 Pa) in the frequency sweep, showed the lowest hardness. GRP-PC, with a G′ of 11,043 Pa, showed intermediate hardness. This apparent contradiction is explained by the different deformation scales: rheology measures network rigidity under small oscillations, whereas TPA quantifies resistance to large-scale compression [31]. The dense crosslinking induced by PC and TA likely creates networks that are rigid under small deformation but brittle under large compression, leading to lower fracture resistance. In contrast, the weaker and less extensive interactions of C and EC produce networks with lower small-deformation rigidity but greater ductility, allowing them to withstand large compression without catastrophic failure.

Thus, TPA complements rheology by providing a direct measure of texture relevant to food application.

### 3.3. Effect of Polyphenols on Simulated In Vitro Digestion of Rice Protein Gels

**Figure 3:**
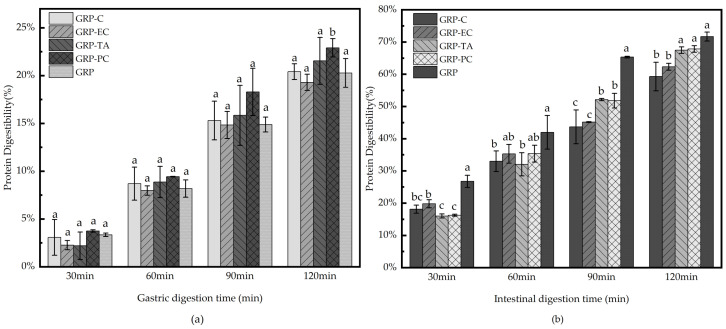
Protein digestibility of rice protein gels during simulated digestion. (**a**) Gastric phase (0–120 min), (**b**) intestinal phase (0–120 min). Note: Different lowercase letters denote significant differences. (*p* < 0.05); GRP: rice protein gel; GRP-C, GRP-EC, GRP-TA, and GRP-PC: the composite gels of rice protein with catechin, epicatechin, tannic acid, and proanthocyanidins, respectively.

The effect of polyphenol–protein composite gels on protein digestibility may involve multiple factors, such as changes in protein structure and alterations in digestive enzyme activity [32]. In the simulated digestion experiment, there were certain differences in the digestibility of gels containing the four polyphenols (Figure 3). The total digestibility in descending order was as follows: GRP (77.43%), GRP-PC (75.22%), GRP-TA (74.49%), GRP-EC (69.59%), and GRP-C (67.61%).

During the simulated gastric digestion phase, the digestibility of each composite gel gradually increased over time, and the values at 120 min of digestion were relatively close: GRP was 20.28%, while the polyphenol-containing gels ranged from 19.30% to 22.91%, among which GRP-PC was slightly higher and GRP-EC was slightly lower. During the simulated intestinal digestion phase, the digestibility of GRP at all time points was higher than that of the polyphenol-containing samples; the digestibility of GRP-TA and GRP-PC in the later stage of digestion (90–120 min) was relatively close to that of GRP, whereas GRP-C and GRP-EC remained consistently lower.

The primary reason for the reduction in protein digestibility upon polyphenol addition is the inhibitory effect of polyphenols on digestive enzymes such as pepsin and trypsin [33,34]. Polyphenols can bind to digestive enzymes, reducing their catalytic activity and thereby slowing down the proteolysis process [35]. The differences in digestibility among the various polyphenols may also be related to their release rate during digestion. As shown in the previous rheological results, GRP-TA and GRP-PC exhibited higher storage moduli, whereas GRP-C and GRP-EC had relatively lower storage moduli. Such differences in gel mechanical properties may affect the release rate of polyphenols from the gel matrix into the digestive fluid, thereby exerting different degrees of inhibition on digestive enzymes.

**Figure 4:**
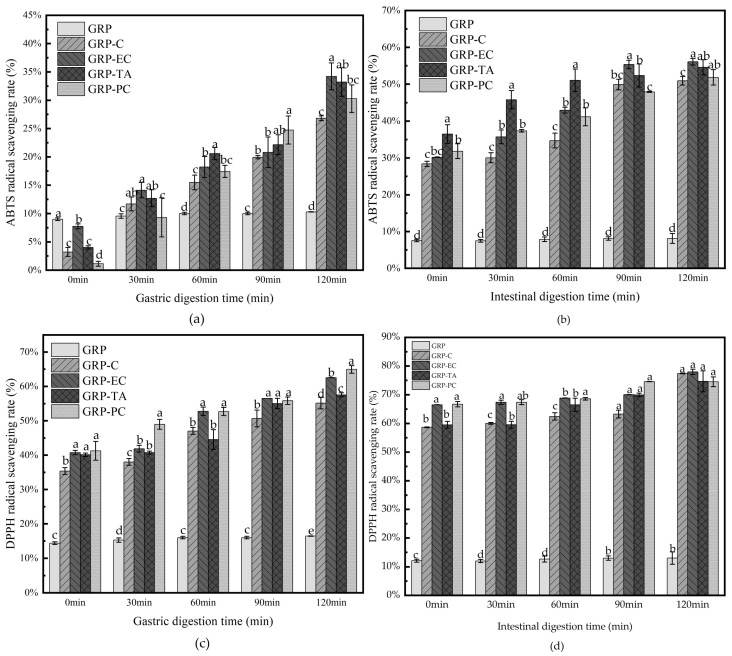
Radical scavenging activity of digesta (120 min) from rice protein gels. (**a**) ABTS, gastric phase; (**b**) ABTS, intestinal phase; (**c**) DPPH, gastric phase; (**d**) DPPH, intestinal phase. Note: Different lowercase letters denote significant differences. (*p* < 0.05); GRP: rice protein gel; GRP-C, GRP-EC, GRP-TA, and GRP-PC: the composite gels of rice protein with catechin, epicatechin, tannic acid, and proanthocyanidins, respectively; ABTS (2,2′-azino-bis(3-ethylbenzothiazoline-6-sulfonic acid)); DPPH (2,2-diphenyl-1-picrylhydrazyl).

As shown in the Figure 4,During the simulated gastric digestion phase, the ABTS scavenging rate of GRP remained consistently low (9.00–10.29%). At 120 min of gastric digestion, all polyphenol-containing gels showed significantly higher ABTS scavenging rates than GRP (10.29%, *p* < 0.05). GRP-EC and GRP-TA were significantly higher than GRP-C (*p* < 0.05).

During the intestinal digestion phase (120 min), the ABTS scavenging rates further increased. All polyphenol-containing samples were significantly higher than GRP (8.12%, *p* < 0.05). GRP-EC and GRP-TA were significantly higher than GRP-C (*p* < 0.05).

The DPPH scavenging rate at 120 min of gastric digestion followed a similar trend: all polyphenol-composite gels were significantly higher than GRP. GRP-EC and GRP-PC exhibited significantly higher values than GRP-C and GRP-TA (*p* < 0.05).

For the DPPH radical scavenging activity in the intestinal phase (120 min), all polyphenol-composite gels were significantly higher than GRP. GRP-TA was significantly lower than GRP-C and GRP-EC (*p* < 0.05), whereas GRP-PC showed no significant difference from any of the other polyphenol groups (*p* > 0.05). The higher radical scavenging activity in the intestinal phase compared to the gastric phase may be attributed to the release of more antioxidant components under intestinal digestion conditions.

### 3.4. Scanning Electron Microscope

**Figure 5:**
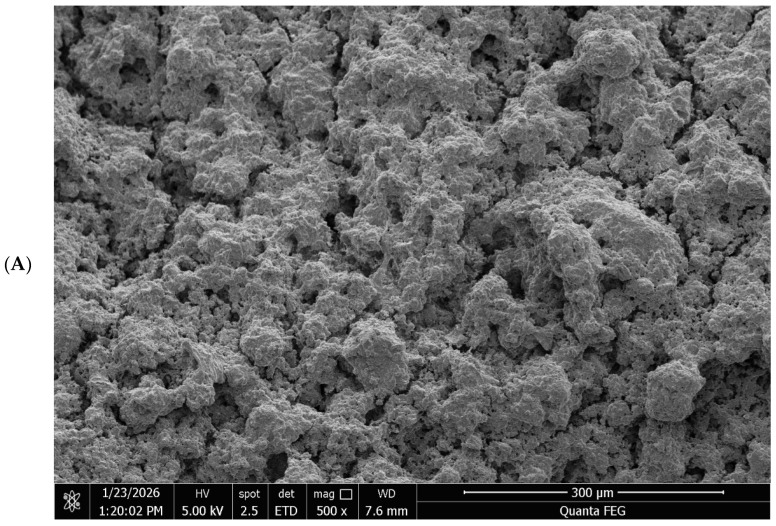

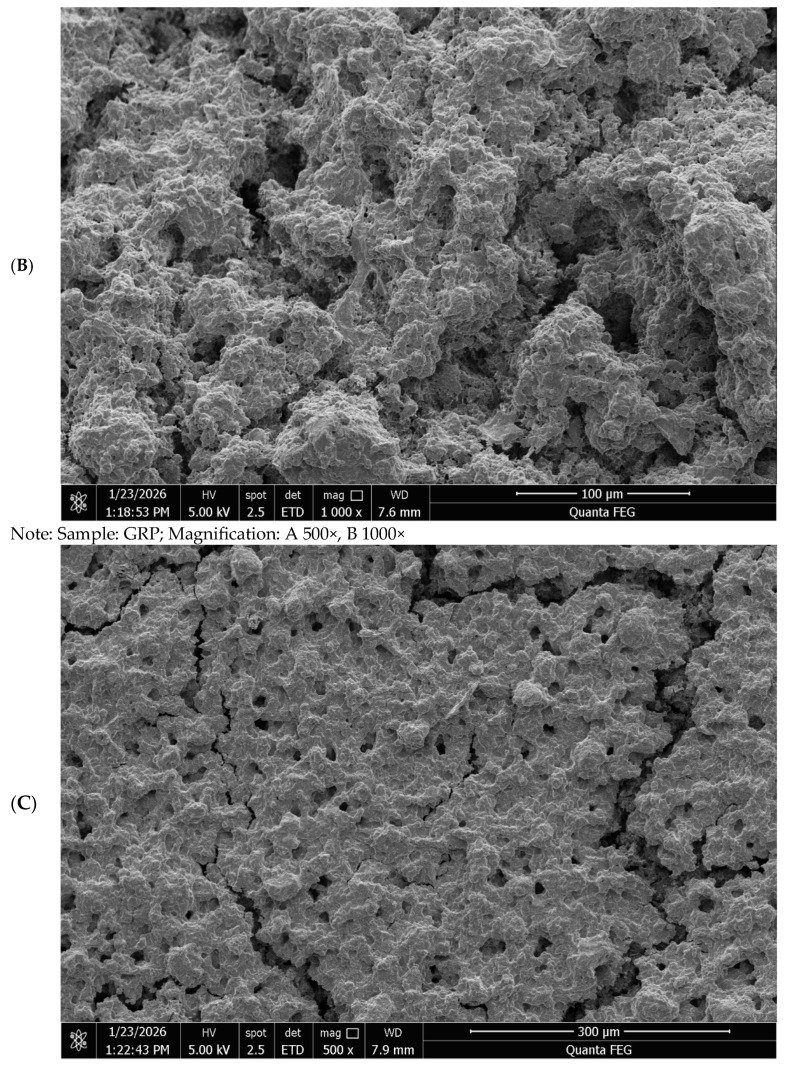

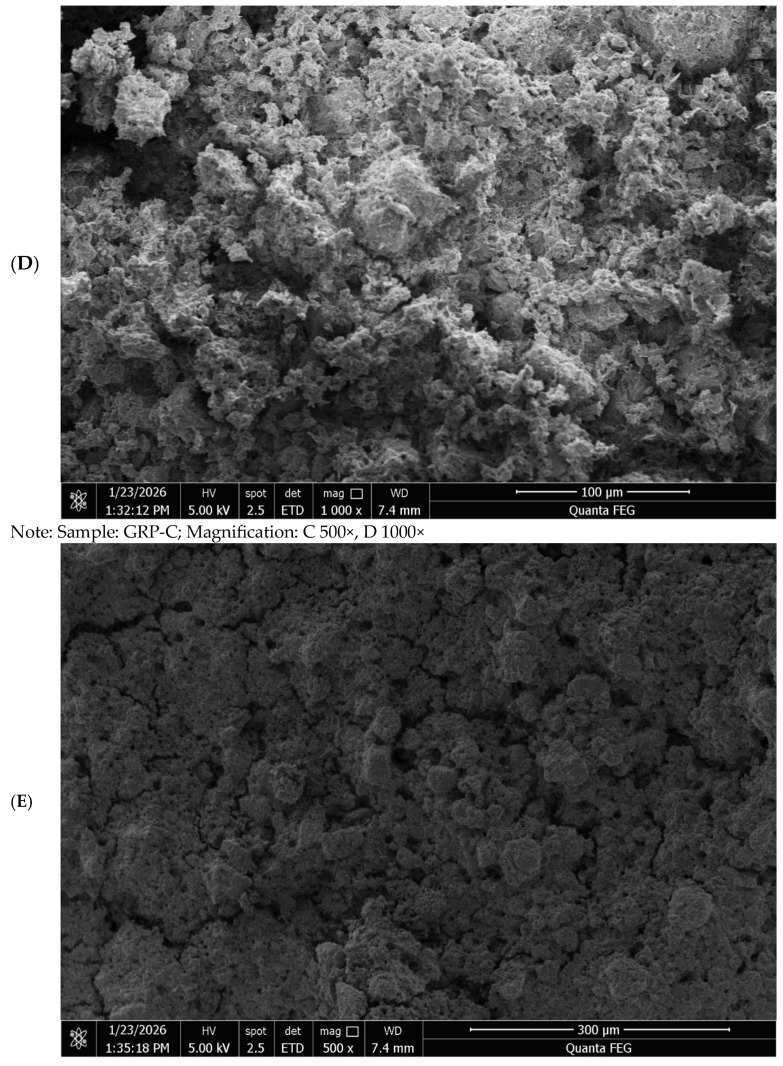

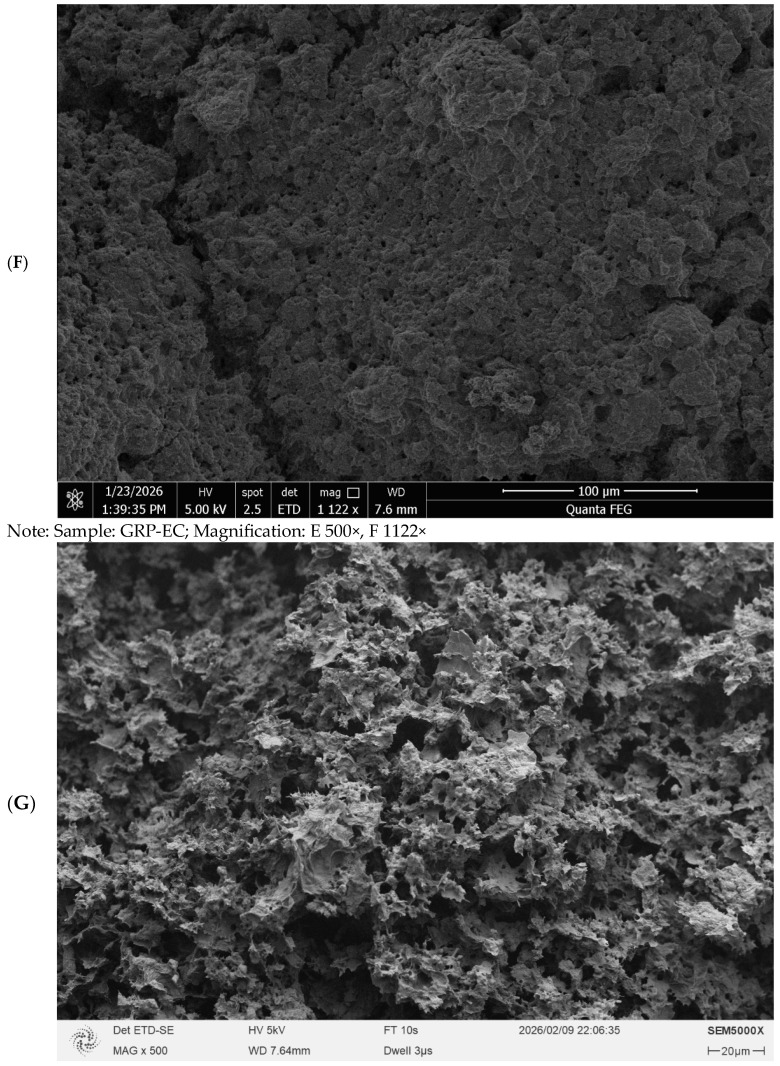

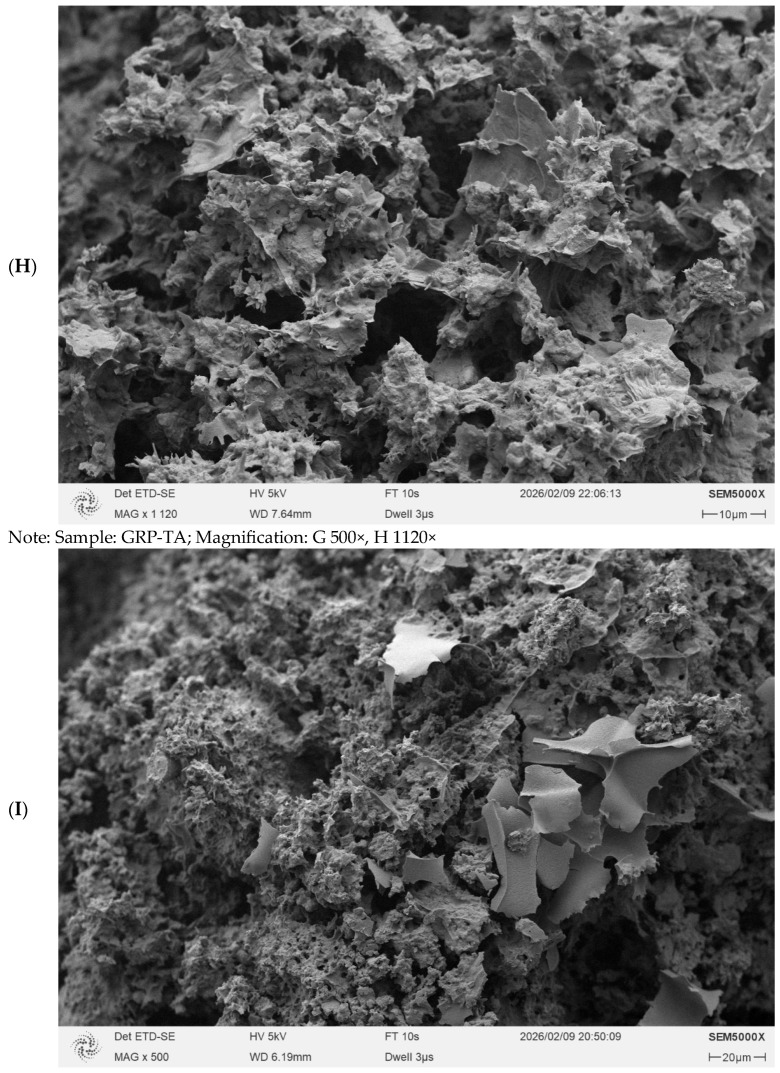

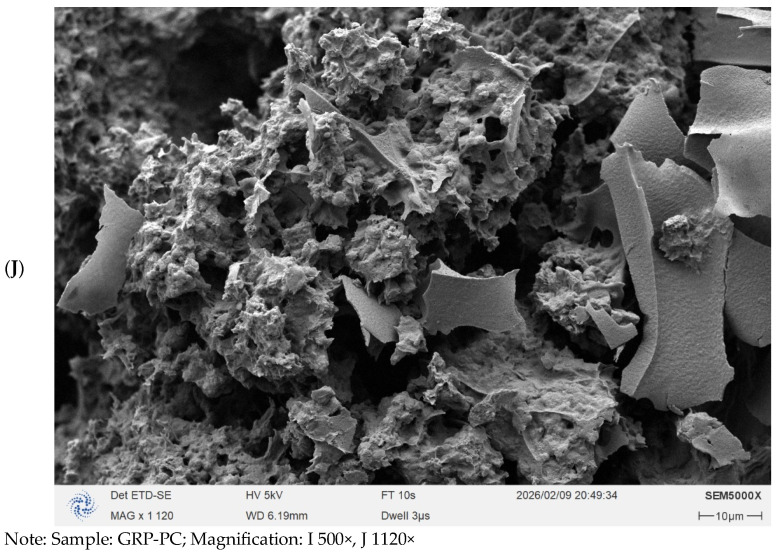
Scanning electron microscopy images of freeze-dried rice protein gels. (**A**) GRP, 500×; (**B**) GRP, 1000×; (**C**) GRP‑C, 500×; (**D**) GRP‑C, 1000×; (**E**) GRP‑EC, 500×; (**F**) GRP‑EC, 1122×; (**G**) GRP‑TA, 500×; (**H**) GRP‑TA, 1120×; (**I**) GRP‑PC, 500×; (**J**) GRP‑PC, 1120×. Scale bars are shown in each image.Note: GRP: pure rice protein gel; GRP‑C, GRP‑EC, GRP‑TA, GRP‑PC: rice protein gels with catechin, epicatechin, tannic acid, and proanthocyanidins, respectively.

Figure 5 shows the scanning electron microscopy (SEM) images of different samples.After freeze-drying, the cross-section of the pure protein gel exhibited a relatively flat microstructure. Under high magnification (1000×), relatively uniformly distributed pores with large diameters were observed, accompanied by slight surface protrusions. This structure indicates that the polyphenol-free protein network was relatively simple and regular. Upon addition of C and EC, the microstructure of the gels changed markedly: the surface became rough, and the pore sizes were unevenly distributed. At 1120× magnification, numerous fine granular substances and a large number of small, dense pores were clearly observed on the surface. C and EC may be distributed within the protein matrix through physical filling or weak binding, causing the network structure to transition from regular to complex. The addition of TA and PC induced more pronounced structural reorganization. Under low magnification (500×), the gel network pores exhibited marked heterogeneity. At high magnification (1120×), a prominent feature was the appearance of numerous sheet-like aggregated structures. Notably, in GRP-PC, these sheet-like structures were even denser. The sheet-like aggregates in GRP-TA and GRP-PC create a rigid but brittle network that resists small-deformation shear (high G′) but exhibits lower fracture resistance under large compression (low TPA hardness). In contrast, the loosely crosslinked, porous structures induced by C and EC allow for more chain mobility and energy dissipation, leading to lower G′ yet comparable or even higher hardness under large deformation.

### 3.5. UV-Vis Analysis

**Figure 6:**
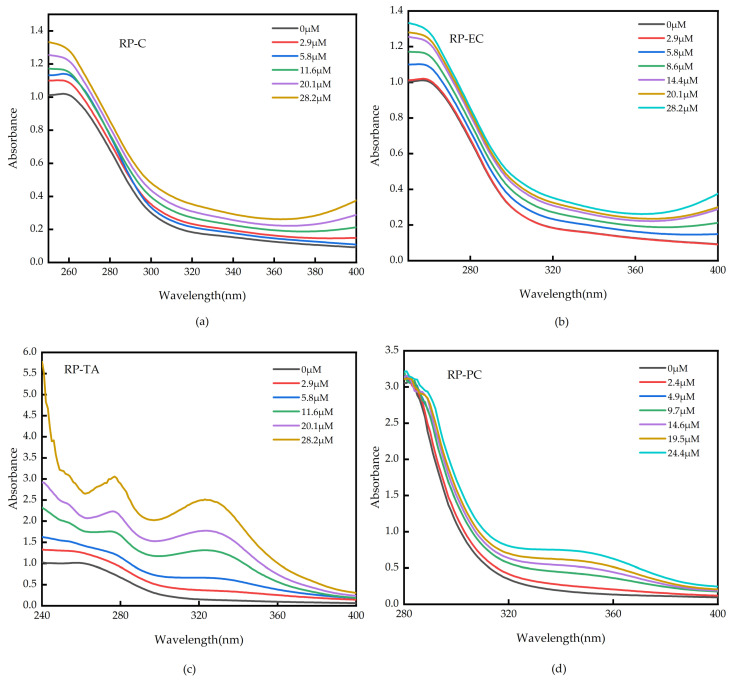
UV-Vis absorption spectra of rice protein (RP) with increasing concentrations of (**a**) catechin (C), (**b**) epicatechin (EC), (**c**) tannic acid (TA), (**d**) proanthocyanidins (PC). Note: The complexes of rice protein with catechin, epicatechin, tannic acid, and proanthocyanidins were designated as RP-C, RP-EC, RP-TA, and RP-PC, respectively.

The interaction between polyphenols and proteins is complex and may involve hydrogen bonding, hydrophobic interactions, π-π stacking, and covalent bond formation [36]. UV-Vis spectroscopy can be used to preliminarily determine whether an interaction occurs between the two. Figure 6 shows the UV absorption spectra of the interactions between the four polyphenols and rice protein. Upon addition of C or EC, the absorbance of RP in the plateau region increased with increasing polyphenol concentration, but the spectral profile remained essentially unchanged, as shown in Figure 6a,b. This indicates that C and EC interacted with RP, altering the microenvironment of the aromatic amino acids in rice protein without causing dramatic conformational rearrangement. C and EC are isomers and exhibited similar trends in their effects on the UV spectra. The addition of TA caused marked changes in the UV spectrum of RP. As shown in Figure 6c, with increasing TA concentration, the original plateau region gradually splits into two distinct absorption peaks, located at 277 nm and 325 nm, respectively. The absorption peak at 277 nm can be attributed to the combined absorption of RP aromatic amino acids and the phenolic hydroxyl groups of TA [37]; the new peak appearing at 325 nm may indicate the formation of a stable ground-state complex [38].

### 3.6. Fluorescence Spectrum

**Figure 7:**
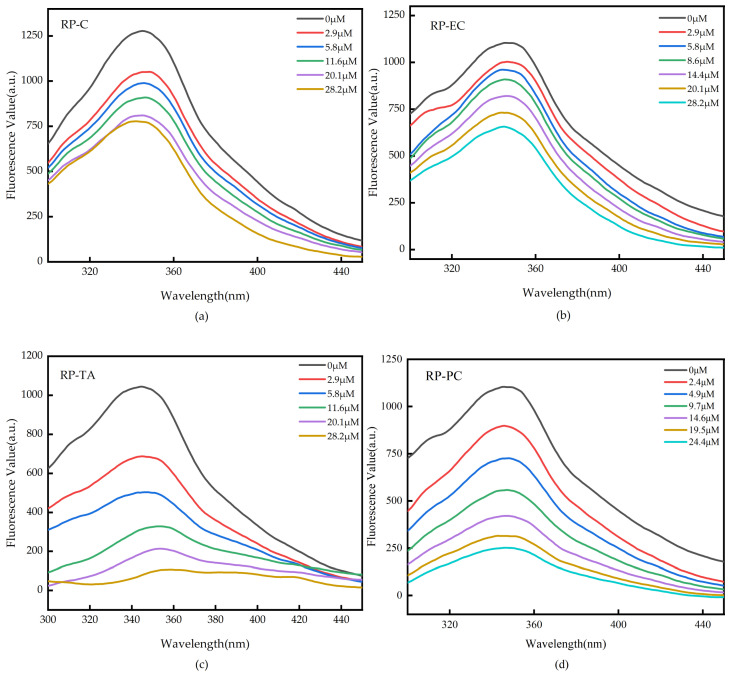
Fluorescence burst spectra of rice protein (RP) with increasing concentrations of (**a**) catechin (C), (**b**) epicatechin (EC), (**c**) tannic acid (TA), (**d**) proanthocyanidins (PC). Note: The complexes of rice protein with catechin, epicatechin, tannic acid, and proanthocyanidins were designated as RP-C, RP-EC, RP-TA, and RP-PC, respectively.

Figure 7 shows the fluorescence quenching spectra of RP after the addition of the four polyphenols at an excitation wavelength of 280 nm (λex = 280 nm), with an absorption peak observed at approximately 345 nm. The maximum absorption wavelength (λmax) in the fluorescence spectrum is related to the microenvironment of tryptophan residues: λmax > 330 nm indicates that tryptophan groups are exposed to a polar environment outside the protein molecule, whereas λmax < 330 nm suggests that tryptophan groups are buried in a nonpolar environment within the protein molecule [39]. After the addition of the four polyphenols, the λmax values were all greater than 330 nm, indicating that the tryptophan residues were mainly located on the exterior of the protein molecule. C and EC caused a blue shift in the absorption peak by 6 nm and 3 nm, respectively, suggesting a change in the protein microenvironment toward hydrophobicity; TA and PC induced a red shift in the RP absorption peak by 14 nm and 1 nm, respectively, indicating a change toward hydrophilicity [40]. The quenching rates of the intrinsic fluorescence of rice protein by the four polyphenols were as follows: TA (89.76%), PC (77.20%), EC (40.59%), and C (39.16%).

### 3.7. Analysis of Quenching Type

The mechanism of fluorescence quenching can be classified into static quenching and dynamic quenching, which are typically distinguished by the temperature dependence of the quenching constant: static quenching (formation of a ground-state complex) is characterized by a decrease in the quenching constant with increasing temperature, whereas dynamic quenching (molecular collision) exhibits an increase with temperature [41]. When both mechanisms coexist, the Stern–Volmer constant (*Ksv*) may still increase with temperature, and the bimolecular quenching constant (*Kq*) often exceeds the maximum diffusion-collision quenching constant (2.0 × 10^10^ L·mol^−1^·s^−1^) [42]. The quenching mechanism in the system can be described by the Stern–Volmer Equation (3):

(3) $$F_{0}/F=Kq\tau_{0}[Q]+1=Ksv[Q]+1$$

where *F*_0_ and *F* are the fluorescence intensities of RP in the absence and presence of polyphenols, respectively; *Kq* is the bimolecular quenching constant; *Ksv* is the Stern–Volmer constant; [*Q*] is the quencher concentration; and τ_0_ is the molecular fluorescence lifetime, taken as 10^−8^ s [43].

**Figure 8:**
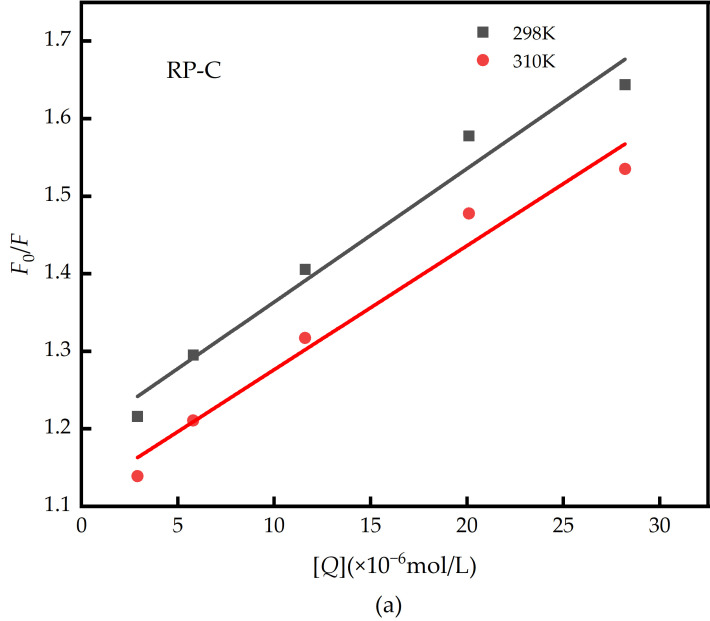

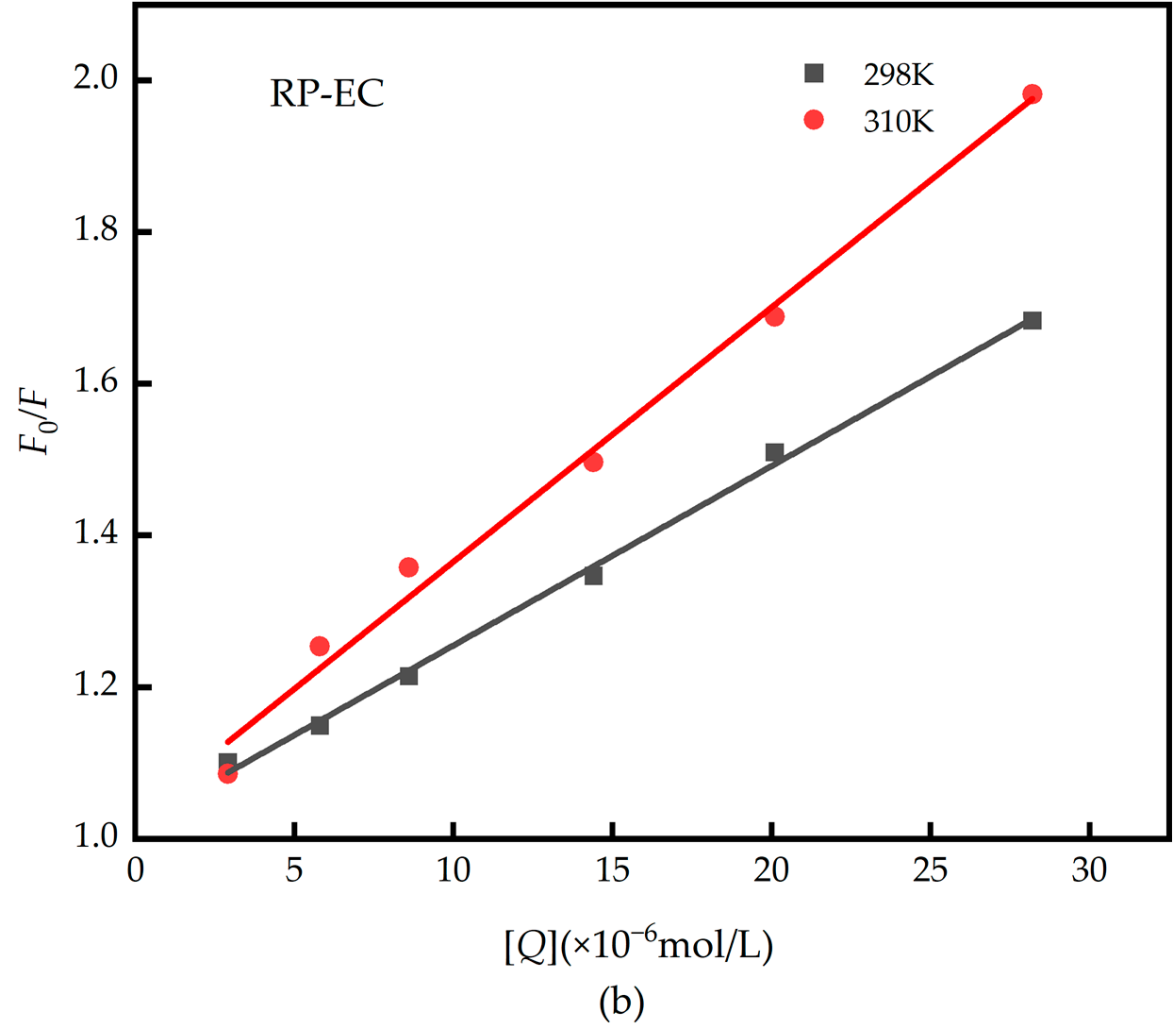

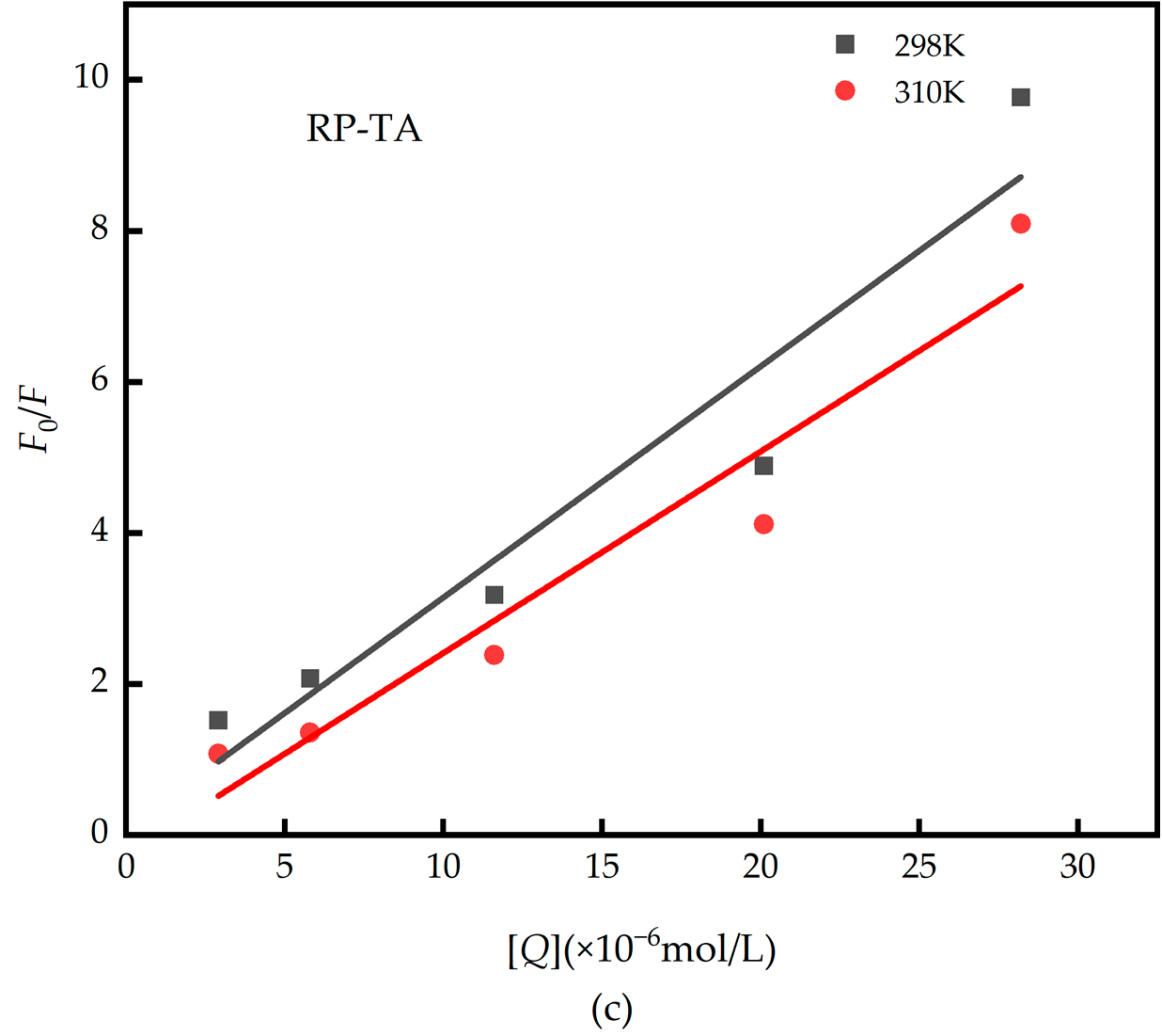

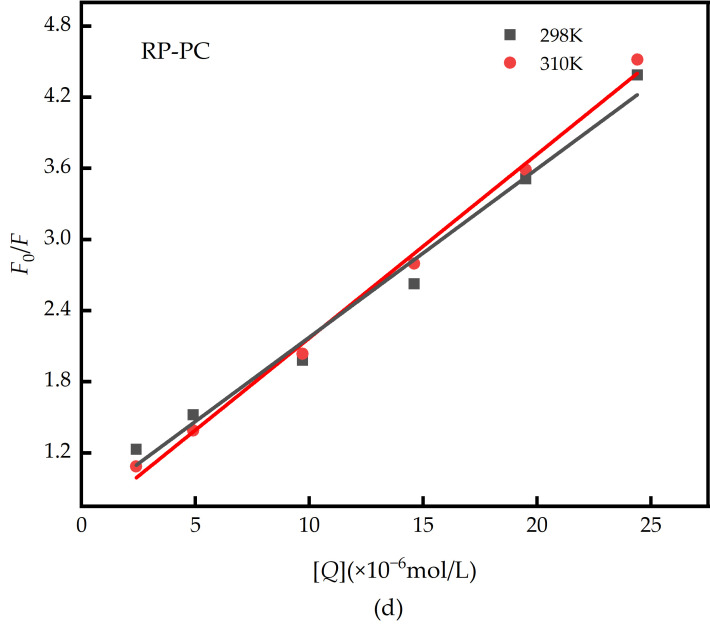
Stern–Volmer plots (F_0_/F vs. [Q]) for the interaction of rice protein (RP) with (**a**) catechin, (**b**) epicatechin, (**c**) tannic acid, and (**d**) proanthocyanidins at 298 K and 310 K. Note: The complexes of rice protein with catechin, epicatechin, tannic acid, and proanthocyanidins were designated as RP-C, RP-EC, RP-TA, and RP-PC, respectively.

Table 2 and Figure 8 show the Stern–Volmer fitting results for the interactions between the four polyphenols and rice protein. The RP-C, RP-EC, and RP-PC systems exhibited good linearity at 298 K and 310 K (*R*^2^ ≥ 0.97), with intercepts close to the theoretical value of 1. The RP-TA system showed relatively lower linearity, with an intercept deviating from 1, suggesting that a single quenching model may not be applicable to this system. The *Kq* values for all systems exceeded 2.0 × 10^10^ L·mol^−1^·s^−1^. Combined with the formation of ground-state complexes observed in the UV-Vis spectra, it can be concluded that static quenching is the predominant quenching mechanism. Further examination of the temperature dependence of *Ksv* revealed that for the RP-C and RP-TA systems, *Ksv* decreased with increasing temperature, consistent with the characteristics of static quenching. In contrast, for the RP-EC and RP-PC systems, *Ksv* increased with temperature, suggesting that molecular motion intensifies at higher temperatures, leading to the coexistence of collisional quenching and static quenching, i.e., a combination of both mechanisms.

### 3.8. Analysis of Binding Constant and Binding Sites

The binding constant (*K*_a_) and the number of binding sites (*n*) can reflect, to a certain extent, the binding level between polyphenols and proteins. The fluorescence quenching data were fitted using a double-logarithmic Equation (4) [44]

(4) $$\mathrm{lg}\left[(F_{0}-F\right)/F]=nlg[Q]+lgK_{a}$$

Equation (5) is the double-logarithmic equation: *F* represents the fluorescence intensity after the addition of polyphenols; *F*_0_ represents the fluorescence intensity of the protein without polyphenols; [*Q*] is the quencher concentration; *K*_a_ and *n* are the binding constant and the number of binding sites, respectively

**Figure 9:**
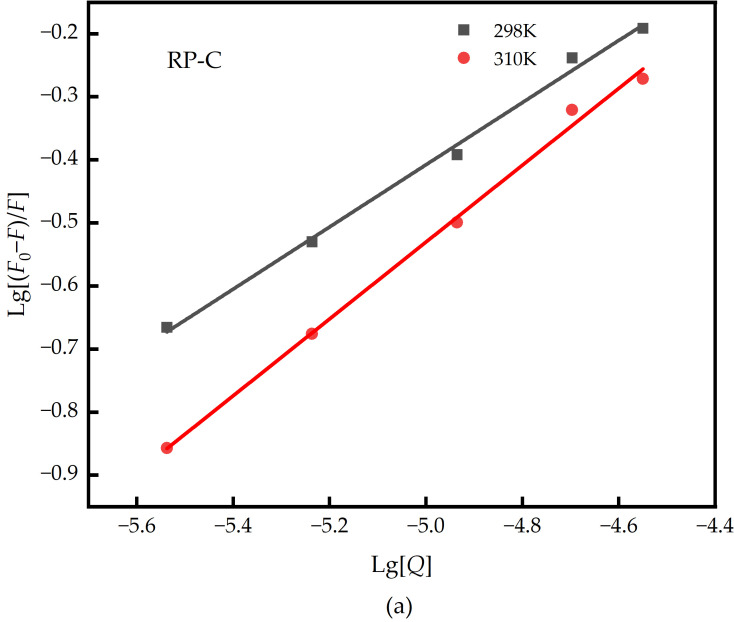

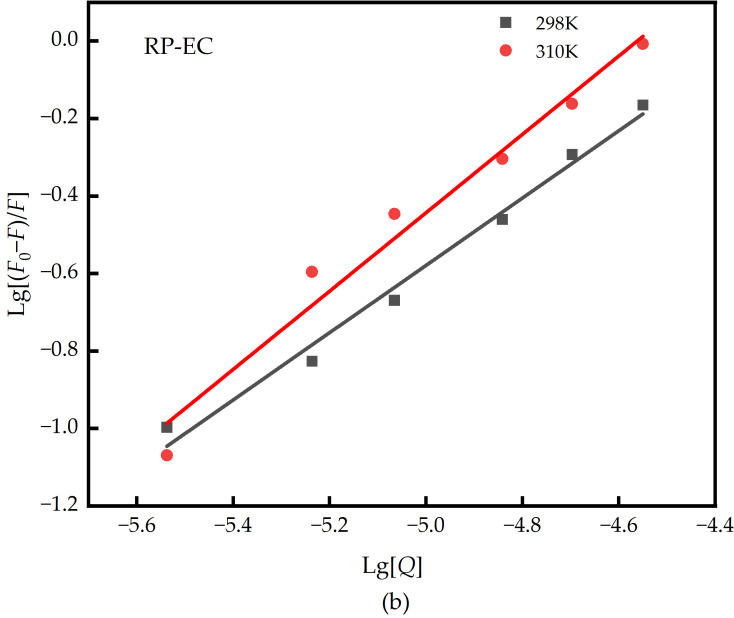

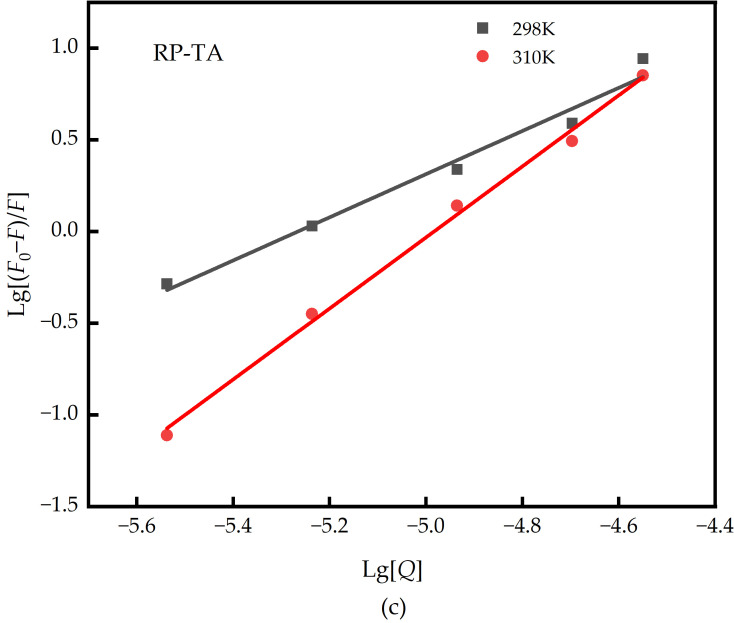

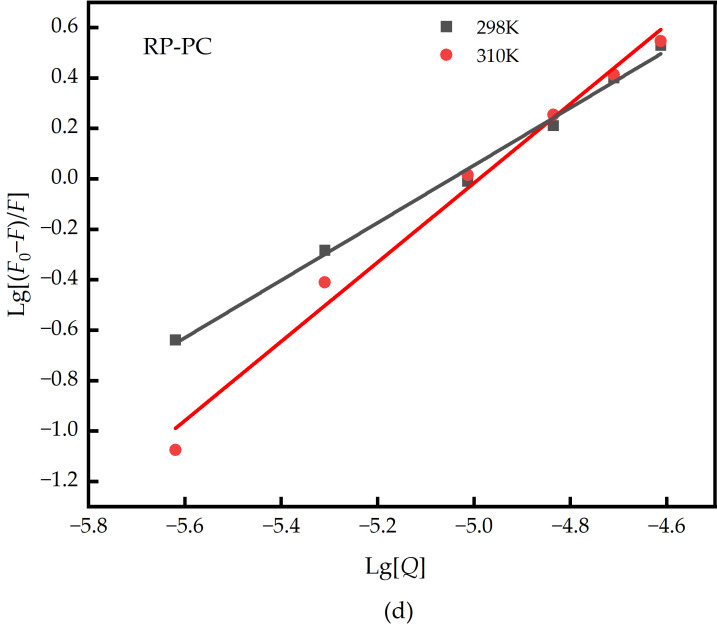
Double-logarithmic plots (lg[(*F*_0_ − *F*)/*F*] vs. lg[*Q*]) for the binding of polyphenols to RP at 310 K. (**a**) RP-C, (**b**) RP-EC, (**c**) RP-TA, (**d**) RP-PC. The binding constants (*K*_a_) and number of binding sites (*n*) were calculated from the intercept and slope. Note: The complexes of rice protein with catechin, epicatechin, tannic acid, and proanthocyanidins were designated as RP-C, RP-EC, RP-TA, and RP-PC, respectively.

Figure 9 and Table 3 present the double-logarithmic curves and parameters for the interactions between the four polyphenols and RP. The binding constant of RP-C was 1.14 × 10^2^ L·mol^−1^, and the number of binding sites *n* = 0.49, which is less than 1. This may be related to the conformational changes induced in the protein upon binding with C, thereby reducing the affinity of subsequent binding sites [45].

As the temperature increased, the binding behavior of the different polyphenols exhibited variations: the number of binding sites for RP-PC remained at 1, while the binding constant increased, indicating that higher temperature favored its binding to the protein. For RP-EC, the number of binding sites was also 1, but the binding constant decreased with increasing temperature. For RP-TA, the number of binding sites increased from 1 to 2, accompanied by an increase in the binding constant. At 310 K, the binding affinities of the four polyphenols to rice protein followed the descending order: TA > PC > EC > C.

### 3.9. Analysis of Non-Covalent Interaction Forces

Non-covalent interactions between polyphenols and proteins mainly include hydrophobic interactions, hydrogen bonds, electrostatic attractions, and van der Waals forces [46]. Thermodynamic parameters, including enthalpy change (Δ*H*), Gibbs free energy change (Δ*G*), and entropy change (Δ*S*), were calculated using the van ‘t Hoff Equations (5) and (6) [47].

(5) $$ln\frac{K2}{K1}=∆H\frac{\frac{1}{T1}-\frac{1}{T2}}{R}$$

(6) ∆G=−RTlnK=∆H−T∆S

In Equations (5) and (6), *K*_1_ and *K*_2_ are the binding constants at temperatures T_1_ and T_2_, respectively; Δ*S* is the entropy change; Δ*H* is the enthalpy change; Δ*G* is the Gibbs free energy change; and R is the gas constant, taken as 8.314 J·mol^−1^·K^−1^.

The data showed that Δ*G* was negative for all systems at both 298 K and 310 K, and the absolute value of Δ*G* increased with temperature, indicating that the binding between polyphenols and rice protein is a spontaneous process and that elevated temperature favors binding. This is consistent with the trend of increasing binding constant (*K*_a_) with temperature.

The Δ*H* and Δ*S* values for all four systems were positive, indicating that the binding process is endothermic and entropy-driven. According to thermodynamic criteria, when Δ*H* > 0 and Δ*S* > 0, the predominant interaction force is hydrophobic interaction. Therefore, the binding of all four polyphenols to the protein is primarily governed by hydrophobic interactions. Further comparison of Δ*H* and Δ*S* among the different systems: the Δ*H* values for RP-C and RP-EC were 67.6 and 125 kJ·mol^−1^, respectively, which fall within the typical range for non-covalent interactions. In contrast, the Δ*H* values for RP-TA and RP-PC were significantly higher, reaching 509.46 and 306.89 kJ·mol^−1^, respectively, consistent with their higher binding constants and greater numbers of binding sites (Table 4). Meanwhile, the Δ*S* values for the RP-TA and RP-PC systems (0.15–0.21 kJ·mol^−1^·K^−1^) were markedly higher than those for the RP-C and RP-EC systems (0.04–0.09 kJ·mol^−1^·K^−1^), indicating stronger hydrophobic interactions and a more pronounced decrease in system order during the binding process for the former two.

### 3.10. Synchronous Fluorescence Spectral Analysis

**Figure 10:**
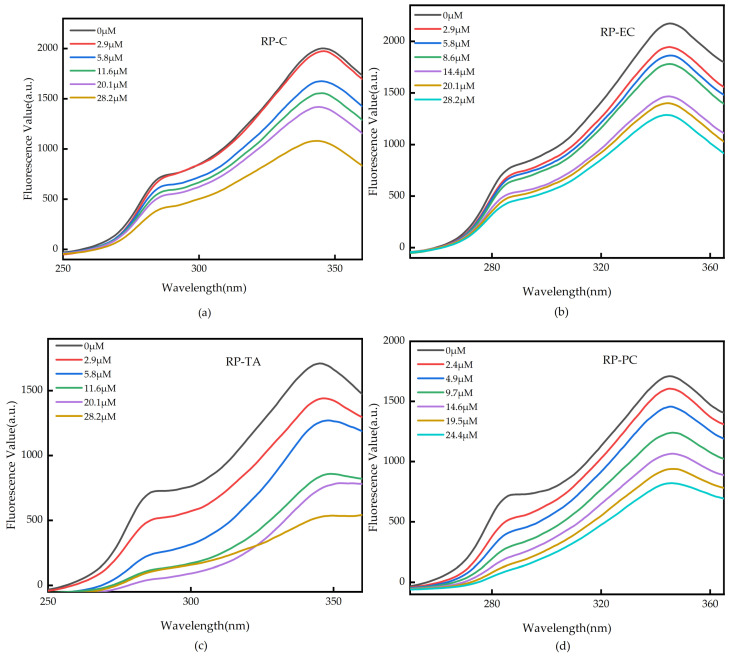
Synchronous fluorescence spectra (Δλ = 15 nm, primarily tyrosine residues) of RP with increasing concentrations of (**a**) C, (**b**) EC, (**c**) TA, and (**d**) PC.

**Figure 11:**
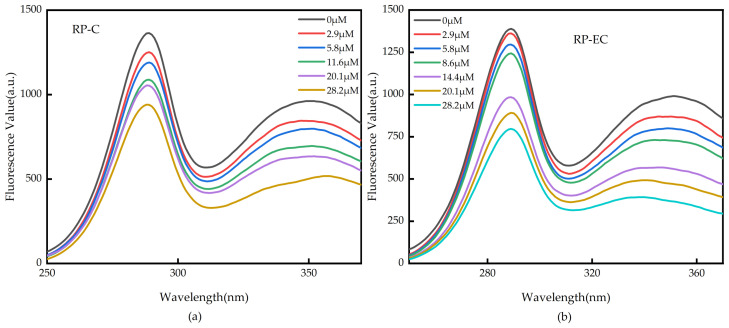

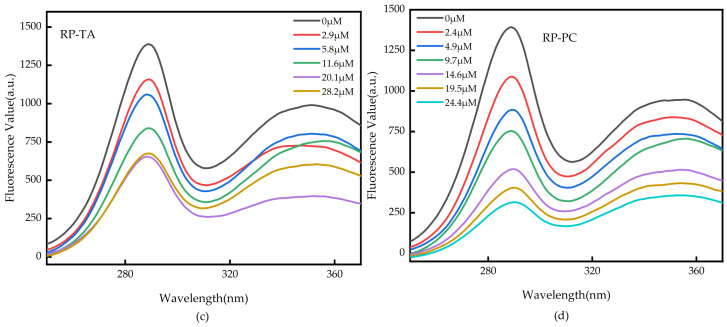
Synchronous fluorescence spectra (Δλ = 60 nm, primarily tryptophan residues) of RP with increasing concentrations of (**a**) C, (**b**) EC, (**c**) TA, and (**d**) PC. Note: The complexes of rice protein with catechin, epicatechin, tannic acid, and proanthocyanidins were designated as RP-C, RP-EC, RP-TA, and RP-PC, respectively.

Figure 10 and Figure 11 show the synchronous fluorescence spectra of the interactions between polyphenols and rice protein at Δλ = 15 nm (primarily reflecting tyrosine residues) and Δλ = 60 nm (primarily reflecting tryptophan residues), respectively. The results indicated that the degree of quenching of the two amino acid residues varied among the different polyphenols: RP-C and RP-TA exhibited stronger quenching of tyrosine than tryptophan, suggesting that the binding sites of these two polyphenols on the protein may be closer to tyrosine residues; in contrast, RP-EC and RP-PC showed stronger quenching of tryptophan, suggesting that their binding sites are closer to tryptophan residues [48]. Furthermore, the addition of all four polyphenols caused a slight red shift in the tryptophan characteristic peak, indicating an increase in the hydrophilicity of the microenvironment around the tryptophan residues.

### 3.11. Three-Dimensional Fluorescence Spectroscopy Analysis

**Figure 12:**
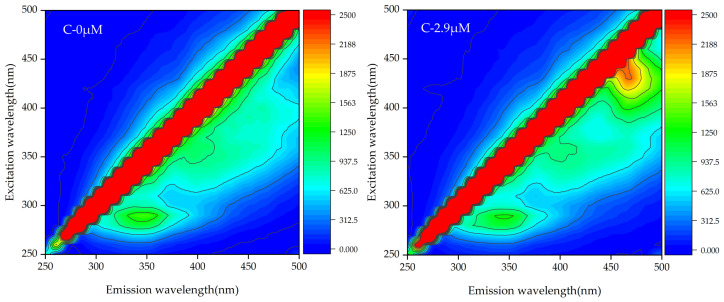

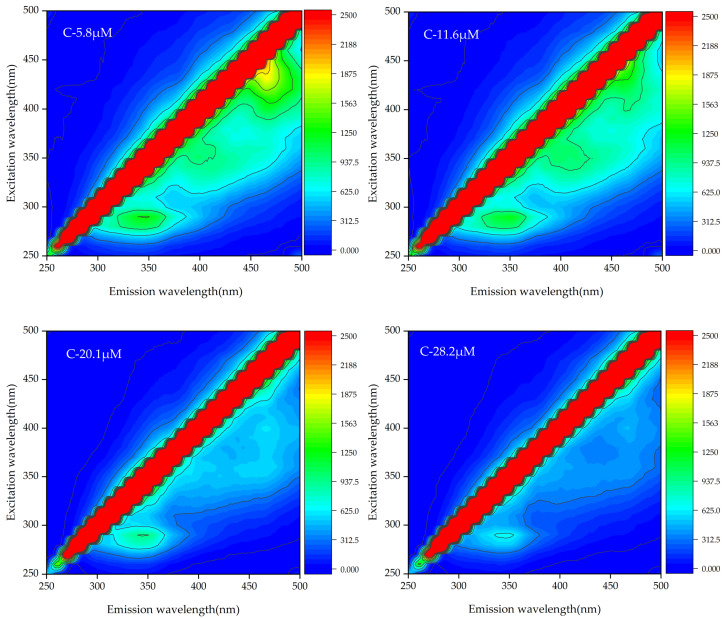
Three-dimensional fluorescence spectra of rice protein with increasing catechin concentrations (0, 2.9, 5.8, 11.6, 20.1, and 28.2 μmol L^−1^). Excitation wavelength 250–500 nm, emission wavelength 250–500 nm.

**Figure 13:**
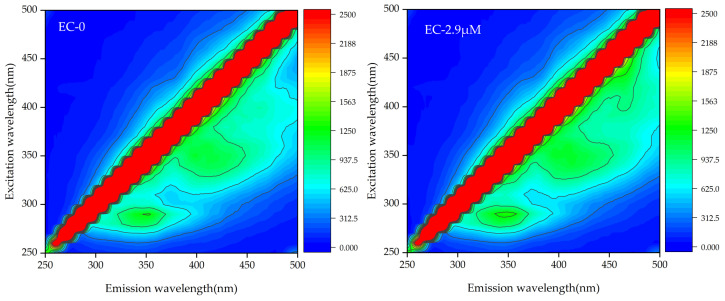

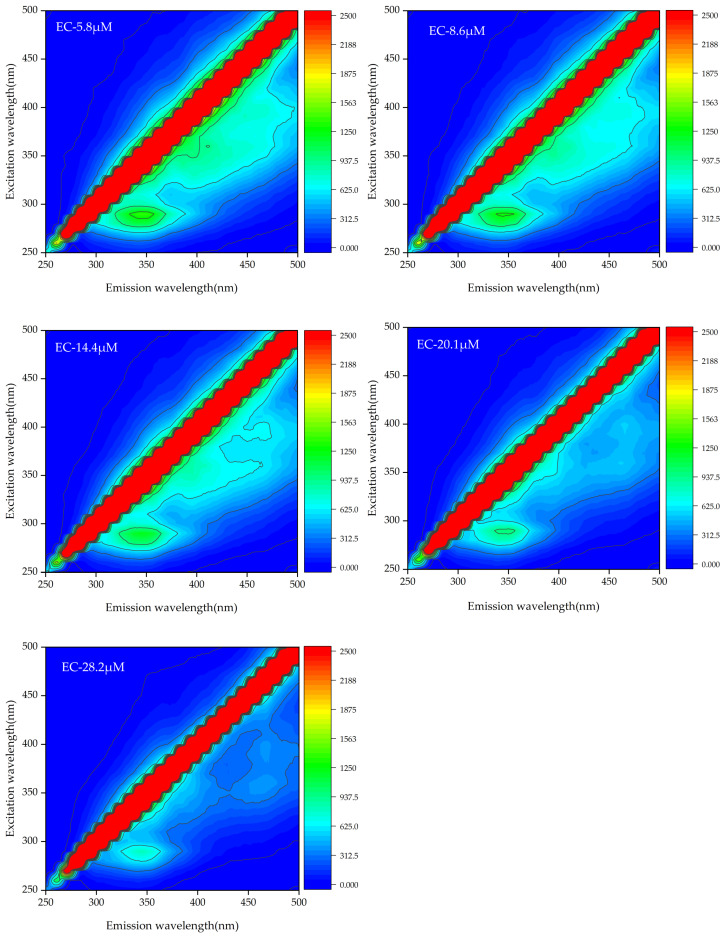
Three-dimensional fluorescence spectra of rice protein with increasing epicatechin concentrations (0, 2.9, 5.8, 8.6, 14.4, 20.1 and 28.2 μmol L^−1^). Excitation wavelength 250–500 nm, emission wavelength 250–500 nm.

**Figure 14:**
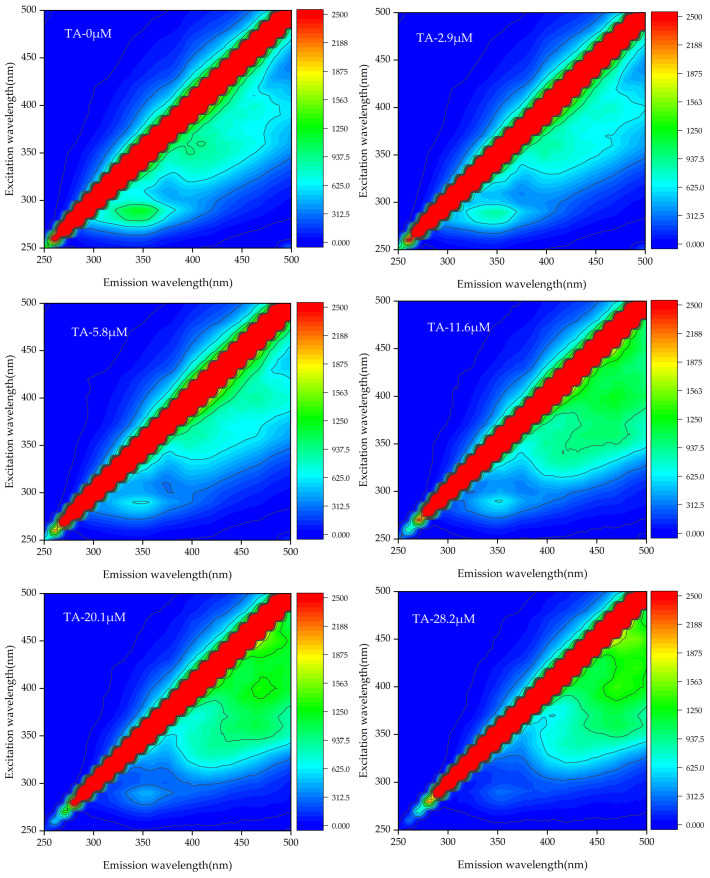
Three-dimensional fluorescence spectra of rice protein with increasing tannic acid concentrations (0, 2.9, 5.8, 11.6, 20.1 and 28.2 μmol L^−1^). Excitation wavelength 250–500 nm, emission wavelength 250–500 nm.

**Figure 15:**
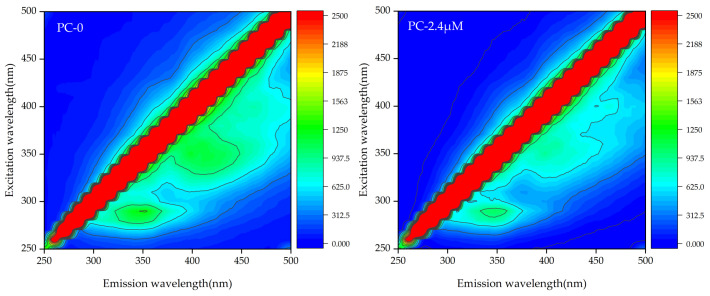

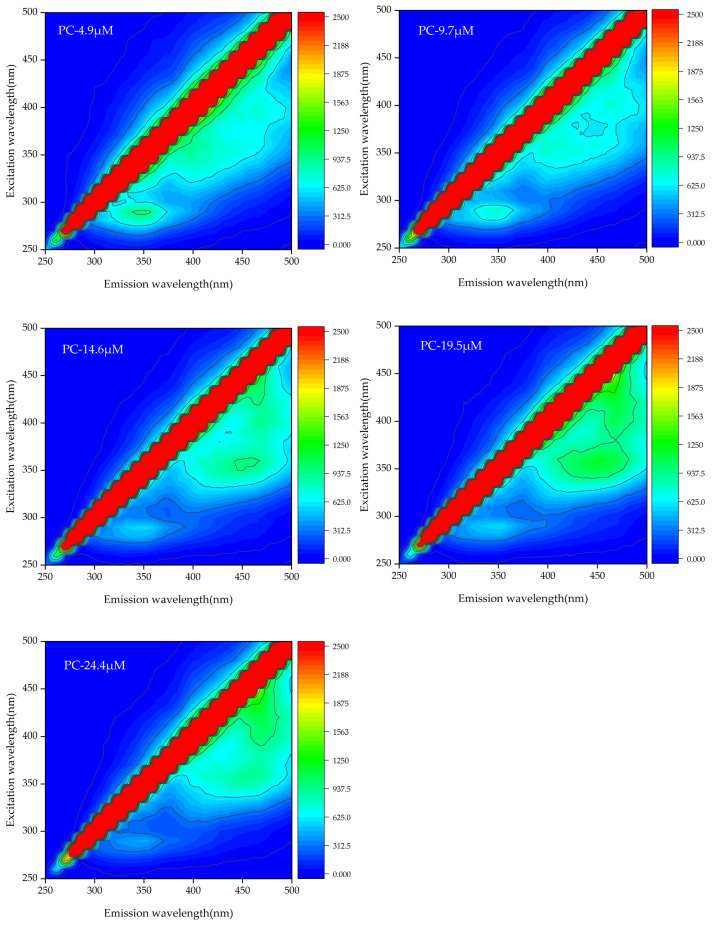
Three-dimensional fluorescence spectra of rice protein with increasing proanthocyanidins concentrations (0, 2.4, 4.9, 9.7, 14.6, 19.5 and 24.4 μmol L^−1^). Excitation wavelength 250–500 nm, emission wavelength 250–500 nm.

Using three-dimensional fluorescence spectroscopy, this study investigated the conformational changes in RP in the presence of varying concentrations of polyphenols [49]. Figure 12, Figure 13, Figure 14 and Figure 15 present the three-dimensional fluorescence spectral characteristics. The fluorescence peak at approximately 350 nm mainly originates from tryptophan residues. With increasing concentrations of the four polyphenols, the fluorescence intensity of this peak gradually decreased, indicating the formation of complexes between the polyphenols and rice protein.

The changes in peak position varied among the different polyphenols: for RP-TA, the fluorescence peak red-shifted from approximately 340 nm to 350 nm with increasing TA concentration, accompanied by a gradual weakening of the peak shape; for RP-PC, the peak position gradually blue-shifted with increasing PC concentration; the changes induced by C and EC were relatively less pronounced. These results indicate that TA and PC exert more pronounced effects on the conformation of RP than C and EC, which may be related to their structural characteristics, such as molecular weight and the number of phenolic hydroxyl groups.

### 3.12. Molecular Docking Simulation of Polyphenols with Rice Glutenin

Molecular docking simulation can be used to predict the binding sites and binding modes of polyphenols with proteins, providing a structural reference for the phenomena observed by spectroscopy [50]. Rice glutelin is the main component of rice protein [51]. In this study, rice glutelin was used as a representative to explore the interaction mechanism between polyphenols and rice protein. The results are shown in Table 5. The minimum binding energies of rice glutelin with catechin, epicatechin, tannic acid, and proanthocyanidins were −5.97, −4.34, −5.94, and −5.76 kcal·mol^−1^, respectively. All values were negative, indicating that these polyphenols can spontaneously form relatively stable complexes with the protein.

**Figure 16:**
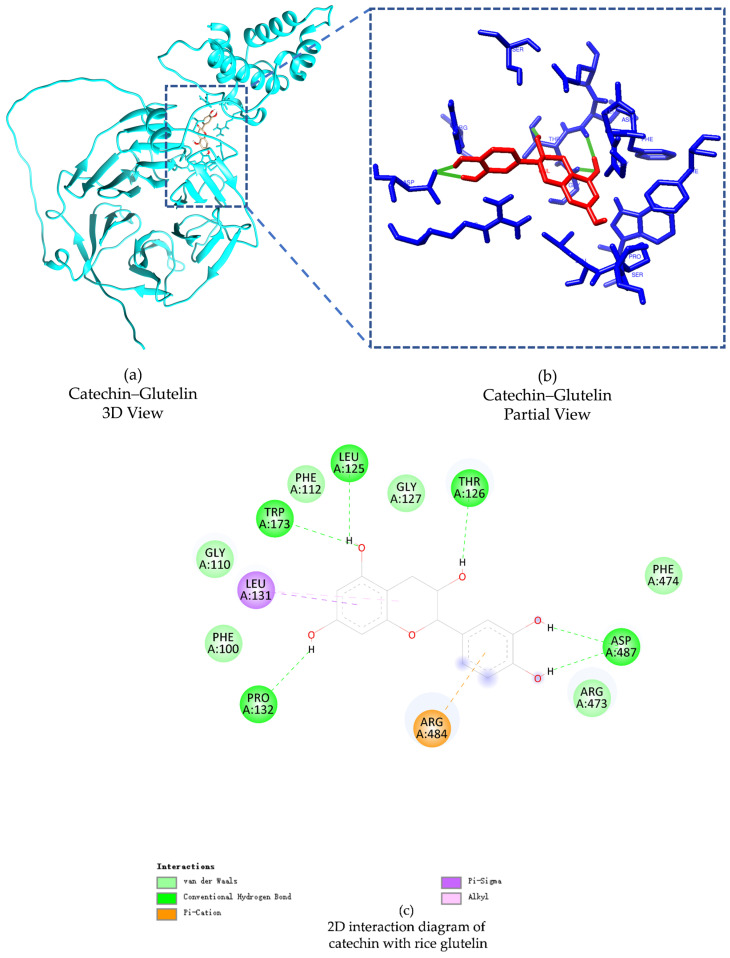
Molecular docking simulation of catechin with rice glutenin. Note: (**a**) 3D diagram; (**b**) partial view; (**c**) 2D map. In (**b**), rice glutelin is shown in blue, catechin in red, and hydrogen bonds as green lines.

**Figure 17:**
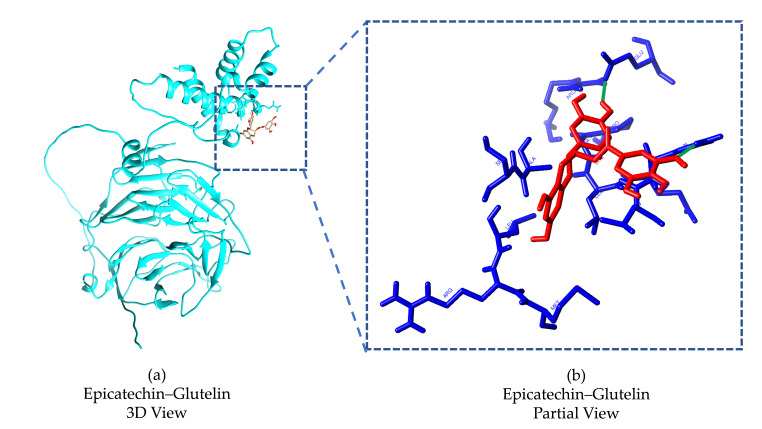

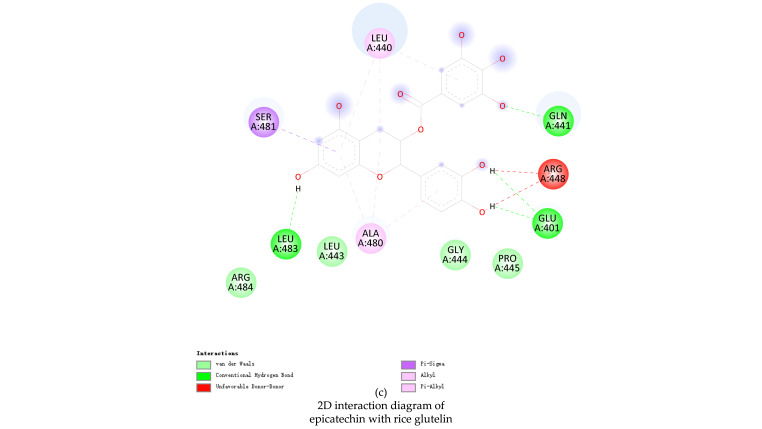
Molecular docking simulation of epicatechin with rice glutelin. Note: (**a**) 3D view; (**b**) local view; (**c**) 2D map. In (**b**), rice glutelin is shown in blue, epicatechin in red, and hydrogen bonds as green lines.

**Figure 18:**
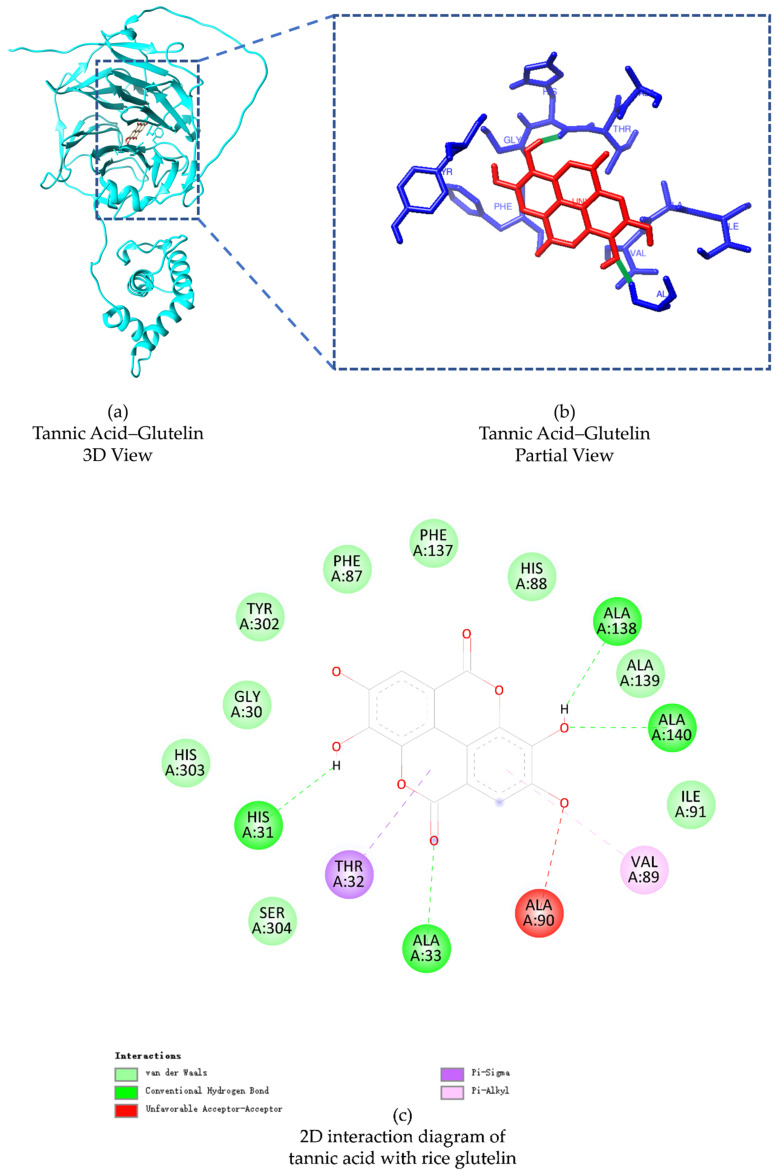
Molecular docking simulation of tannic acid with rice glutelin. Note: (**a**) 3D view; (**b**) local view; (**c**) 2D map. In (**b**), rice glutelin is shown in blue, tannic acid in red, and hydrogen bonds as green lines.

**Figure 19:**
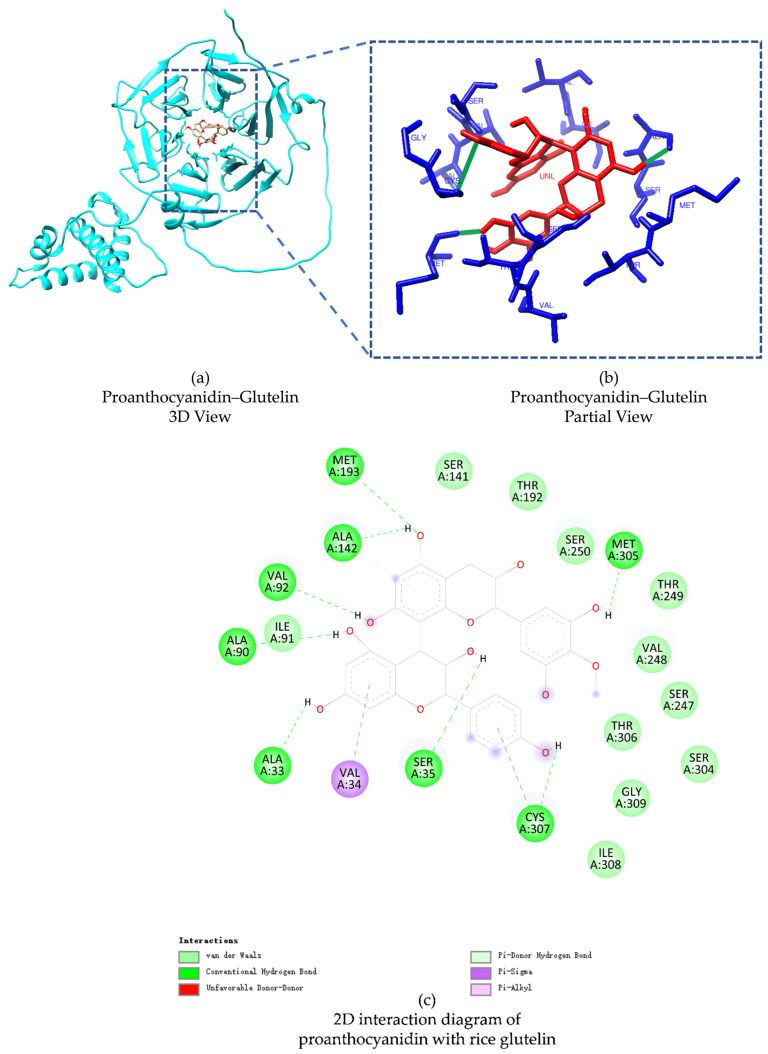
Molecular simulation docking of proanthocyanidins with rice gluten. Note: (**a**) 3D view; (**b**) local view; (**c**) 2D map. In (**b**), rice glutelin is shown in blue, proanthocyanidins in red, and hydrogen bonds as green lines.

Figure 16, Figure 17, Figure 18 and Figure 19 illustrate the differences in the number of hydrogen bonds formed between the different polyphenols and rice glutelin: catechin formed five hydrogen bonds, epicatechin formed three, tannic acid formed four, and proanthocyanidins formed eight. The molecular docking results support the differences in binding ability among the various polyphenols with rice protein.

The greater hydrogen-bonding capacity of proanthocyanidins (eight bonds) and tannic acid (four bonds) is consistent with their larger molecular size and abundant phenolic hydroxyl groups. These extensive hydrogen bonds are likely to contribute to the higher cross-linking density observed in PC and TA gels, which underlie their higher storage moduli (Section 3.1). Notably, catechin formed five hydrogen bonds but did not enhance gel modulus or binding affinity, suggesting that static hydrogen bond count alone does not guarantee strong or effective crosslinking; the spatial arrangement and binding geometry are equally important. For tannic acid (an ester-linked hydrolysable tannin) and proanthocyanidins (a carbon–carbon-linked oligomer), the different hydrogen-bonding patterns may offer a rationalization for their distinct TPA behaviors: the flexible ester linkages in TA could lead to uneven stress distribution and brittle failure, whereas the rigid C-C backbone of PC might be associated with a more resilient network. Collectively, the docking results provide a possible molecular-level interpretation for the observed macroscopic property differences among the four polyphenols.

## 4. Conclusions

All four polyphenols (catechin, epicatechin, tannic acid, and proanthocyanidins) affected the rheological behavior, digestibility, and microstructure of rice protein gels, and the interaction modes between the four polyphenols and rice protein differed. The main conclusions are as follows:

(1) At the end of the temperature sweep (95 °C), the storage moduli of GRP-PC, GRP-TA, and GRP were comparable and nearly superimposable, while GRP-EC and GRP-C were distinctly lower. In contrast, after cold storage, the frequency sweep (25 °C) showed that GRP had a much higher modulus (20,670 Pa) than all polyphenol-composite gels, indicating that the pure protein network can rearrange more freely during refrigeration, whereas the crosslinks introduced by polyphenols, especially PC and TA, restrict chain mobility and result in a more shear-sensitive structure.
(2) Digestibility was reduced by all polyphenols. C caused the strongest reduction (from 77.4% to 67.6%, a decrease of 9.8%), followed by EC (to 69.6%, a decrease of 7.8%), while PC and TA caused only minor reductions (to 75.2% and 74.5%, respectively). The stronger inhibitory effect of C and EC is likely due to their weaker binding to rice protein, allowing rapid release from the gel matrix and early inhibition of digestive enzymes. In contrast, all polyphenol-containing gels exhibited markedly increased radical scavenging activity; EC and PC showed the highest DPPH scavenging rates (77.99% and 76.51% in the intestinal phase, respectively), more than six times that of GRP (12.98%).
(3) Microstructurally, C and EC induced loosely crosslinked, porous networks, whereas TA and PC formed dense, sheet-like aggregates. Fluorescence quenching and thermodynamic analysis indicated static, spontaneous binding driven by hydrophobic interactions (ΔH > 0, ΔS > 0, ΔG < 0). Molecular docking showed that PC formed 8 hydrogen bonds with rice glutelin, TA 4, C 5, and EC 3.

These findings provide a basis for selecting polyphenols according to desired functionalities: EC for antioxidant-enriched gels, C for controlled protein digestibility, and PC or TA for high crosslinking density during thermal gelation.

## Figures and Tables

**Table 1:** TPA parameters of different rice protein gels.

Sample	Hardness (N)	Gumminess (N)	Chewiness (N)	Springiness	Cohesiveness	Resilience
GRP	2.35 ± 0.06 ^a^	0.87 ± 0.02 ^a^	0.81 ± 0.02 ^a^	0.92 ± 0.003 ^b^	0.37 ± 0.001 ^a^	0.04 ± 0.001 ^b^
GRP-C	2.29 ± 0.04 ^a^	0.81 ± 0.01 ^b^	0.79 ± 0.01 ^a^	0.98 ± 0.023 ^a^	0.35 ± 0.002 ^a^	0.05 ± 0.001 ^a^
GRP-EC	2.06 ± 0.03 ^b^	0.78 ± 0.01 ^b^	0.77 ± 0.01 ^a^	0.98 ± 0.005 ^a^	0.38 ± 0.005 ^a^	0.05 ± 0.001 ^a^
GRP-TA	1.33 ± 0.13 ^d^	0.33 ± 0.05 ^c^	0.25 ± 0.04 ^c^	0.78 ± 0.009 ^c^	0.24 ± 0.031 ^c^	0.03 ± 0.005 ^c^
GRP-PC	1.57 ± 0.03 ^c^	0.49 ± 0.02 ^a^	0.49 ± 0.02 ^b^	1 ± 0.001 ^a^	0.31 ± 0.006 ^b^	0.04 ± 0.001 ^b^

Note: Different superscript letters within the same row indicate significant differences among samples (*p* < 0.05). GRP: rice protein gel; GRP-C, GRP-EC, GRP-TA, and GRP-PC: the composite gels of rice protein with catechin, epicatechin, tannic acid, and proanthocyanidins, respectively.

**Table 2:** Stern–Volmer quenching constants (*Ksv*), bimolecular quenching constants (*Kq*), and correlation coefficients (*R*^2^) for the interaction of polyphenols with rice protein at 298 K and 310 K.

Sample	T/K	SV Equation	*Ksv* (10^6^ L·mol^−1^)	*Kq* (10^14^ L·mol^−1^·s^−1^)	*R* ^2^
RP-C	298	*F*_0_/*F* = 0.0172[*Q*] + 1.1918	0.0172	0.0172	0.97
	310	*F*_0_/*F* = 0.0160[*Q*] + 1.1165	0.016	0.016	0.97
RP-EC	298	*F*_0_/*F* = 0.0237[*Q*] + 1.0185	0.0237	0.0237	0.99
	310	*F*_0_/*F* = 0.0335[*Q*] + 1.0301	0.0335	0.0335	0.99
RP-TA	298	*F*_0_/*F* = 0.3060[*Q*] + 0.0869	0.306	0.306	0.92
	310	*F*_0_/*F* = 0.2668[*Q*] − 0.2565	0.2668	0.2668	0.93
RP-PC	298	*F*_0_/*F* = 0.1420[*Q*] + 0.7549	0.142	0.142	0.98
	310	*F*_0_/*F* = 0.1551[*Q*] + 0.6167	0.1551	0.1551	0.99

Note: The complexes of rice protein with catechin, epicatechin, tannic acid, and proanthocyanidins were designated as RP-C, RP-EC, RP-TA, and RP-PC, respectively.

**Table 3:** Binding constants (*K_a_*, L·mol^−1^), number of binding sites (*n*), and correlation coefficients (*R*^2^) for the interaction of rice protein (RP) with catechin (C), epicatechin (EC), tannic acid (TA), and proanthocyanidins (PC) at 298 K and 310 K.

Sample	T/K	Double Logarithmic Equation	*K_a_*	*n*	*R* ^2^
RP-C	298	lg[(*F*_0_ − *F*)/*F*] = 0.493lg[*Q*] + 2.0571	1.14 × 10^2^	0.493	0.99
	310	lg[(*F*_0_ − *F*)/*F*] = 0.6092lg[*Q*] + 2.5158	3.28 × 10^2^	0.6092	0.99
RP-EC	298	lg[(*F*_0_ − *F*)/*F*] = 0.8682lg[*Q*] + 3.7622	5.78 × 10^3^	0.8682	0.99
	310	lg[(*F*_0_ − *F*)/*F*] = 1.0107lg[*Q*] + 4.6104	4.08 × 10^4^	1.0107	0.97
RP-TA	298	lg[(*F*_0_ − *F*)/*F*] = 1.1761lg[*Q*] + 6.1938	1.56 × 10^6^	1.1761	0.98
	310	lg[(*F*_0_ − *F*)/*F*] = 1.9366lg[*Q*] + 9.6507	4.47 × 10^9^	1.9366	0.99
RP-PC	298	lg[(*F*_0_ − *F*)/*F*] = 1.1395lg[*Q*] + 5.7516	5.64 × 10^5^	1.1395	0.99
	310	lg[(*F*_0_ − *F*)/*F*] = 1.5701lg[*Q*] + 7.834	6.82 × 10^7^	1.5701	0.99

Note: The complexes of rice protein with catechin, epicatechin, tannic acid, and proanthocyanidins were designated as RP-C, RP-EC, RP-TA, and RP-PC, respectively.

**Table 4:** Thermodynamic parameters (Δ*H*, Δ*S*, Δ*G*) for the interaction of rice protein with catechin (C), epicatechin (EC), tannic acid (TA), and proanthocyanidins (PC) at 298 K and 310 K.

Sample	T/K	Δ*H* (kJ·mol^−1^)	Δ*G* (kJ·mol^−1^)	Δ*S* (kJ·mol^−1^·K^−1^)
RP-C	298	67.6	−11.74	0.04
	310		−14.93	0.05
RP-EC	298	125	−21.46	0.08
	310		−27.36	0.09
RP-TA	298	509.46	−35.33	0.15
	310		−57.27	0.21
RP-PC	298	306.89	−32.81	0.13
	310		−46.49	0.17

Note: The complexes of rice protein with catechin, epicatechin, tannic acid, and proanthocyanidins were designated as RP-C, RP-EC, RP-TA, and RP-PC, respectively.

**Table 5:** Minimum binding energies (kcal·mol^−1^) from molecular docking of catechin (C), epicatechin (EC), tannic acid (TA), and proanthocyanidins (PC) with rice glutelin.

Sample	Minimum Binding Energy (kcal·mol^−1^)
Rice Gluten–Catechin	−5.97
Rice Gluten–Epicatechin	−4.34
Rice Gluten–Tannic Acid	−5.94
Rice Gluten–Proanthocyanidins	−5.76

## Data Availability

The original contributions presented in the study are included in the article; further inquiries can be directed to the corresponding author.

## Supplementary Materials

The following supporting information can be downloaded at: https://www.mdpi.com/article/10.3390/foods15111854/s1, Figure S1: The effect of polyphenol-protein interaction on Fourier transform infrared spectroscopy.
