# Supplementary material for: Study on the Effects of Polyphenols on the Properties, Microstructure, and Digestibility of Rice Protein Gel and the Interaction Mechanisms Between Polyphenols and Rice Protein
Source: Foods. 2026 May 24;15(11):1854. doi: 10.3390/foods15111854 (PMC13256330; doi:10.3390/foods15111854)
Supplement: Supplementary file 1 [file foods-15-01854-s001.zip › FTIR.pdf]

FTIR measurement

Freeze-dried polyphenol-protein gel powder was mixed with KBr at a ratio of 1:200 and ground together, then pressed into translucent pellets. FTIR spectra were recorded in transmission mode using a Nicolet FTIR spectrometer (Thermo Fisher Scientific, USA) in the range of 4000–400 cm^{-1} with a resolution of 4 cm^{-1} and 64 scans per sample. A background spectrum (air) was collected before each sample and automatically subtracted. All measurements were performed at room temperature (25 °C). The raw spectra were baseline-corrected and deconvoluted using OMNIC software. Second derivative and Fourier self-deconvolution were applied to resolve the overlapped amide I band (1600–1700 cm^{-1}). Curve fitting was performed with a Gaussian function using Peakfit v4.12 software. The fitted sub-peaks were assigned to secondary structures according to literature[1] (α-helix ~1650 cm^{-1}, β-sheet ~1630 and ~1690 cm^{-1}, β-turn ~1670 cm^{-1}, random coil ~1640 cm^{-1}). The relative contents of protein secondary structures were calculated from the integrated areas of the fitted sub-peaks.

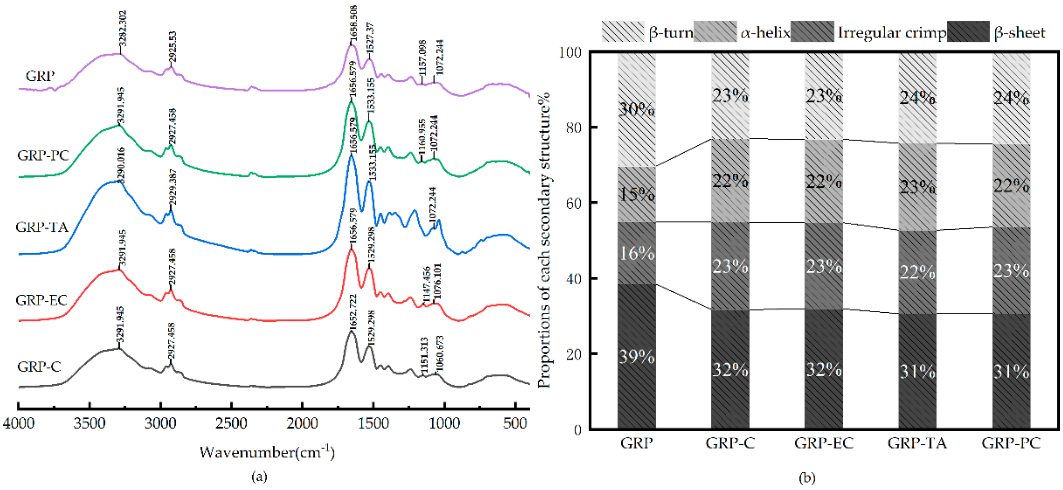

Figure S1 The effect of polyphenol-protein interaction on Fourier transform infrared spectroscopy(a: Functional groups, b: Protein secondary structure)

Reference

1. Yang, Q.; Wang, Y.-R.; Li-Sha, Y.-J.; Chen, H.-Q. Physicochemical, Structural and Gelation Properties of Arachin-Basil Seed Gum Composite Gels: Effects of Salt Types and Concentrations. *Food Hydrocolloids* **2021**, *113*, 106545, doi:10.1016/j.foodhyd.2020.106545.
