# Supplementary figures and images for: Study on the Effects of Polyphenols on the Properties, Microstructure, and Digestibility of Rice Protein Gel and the Interaction Mechanisms Between Polyphenols and Rice Protein
Source: Foods. 2026 May 24;15(11):1854. doi: 10.3390/foods15111854 (PMC13256330; doi:10.3390/foods15111854)

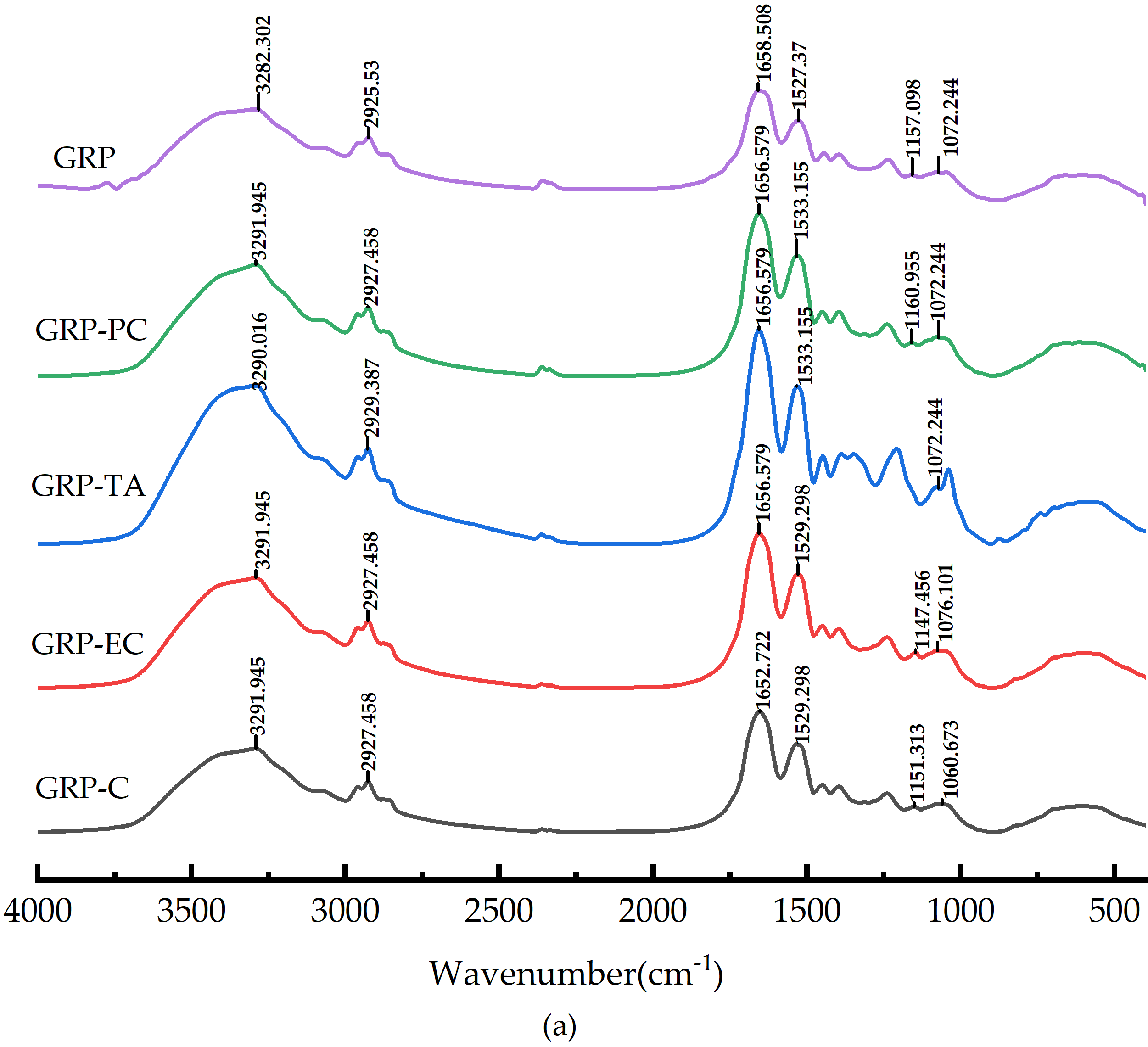

Supplement: Supplementary file 1 [file foods-15-01854-s001.zip › FigureS1-1.tif]

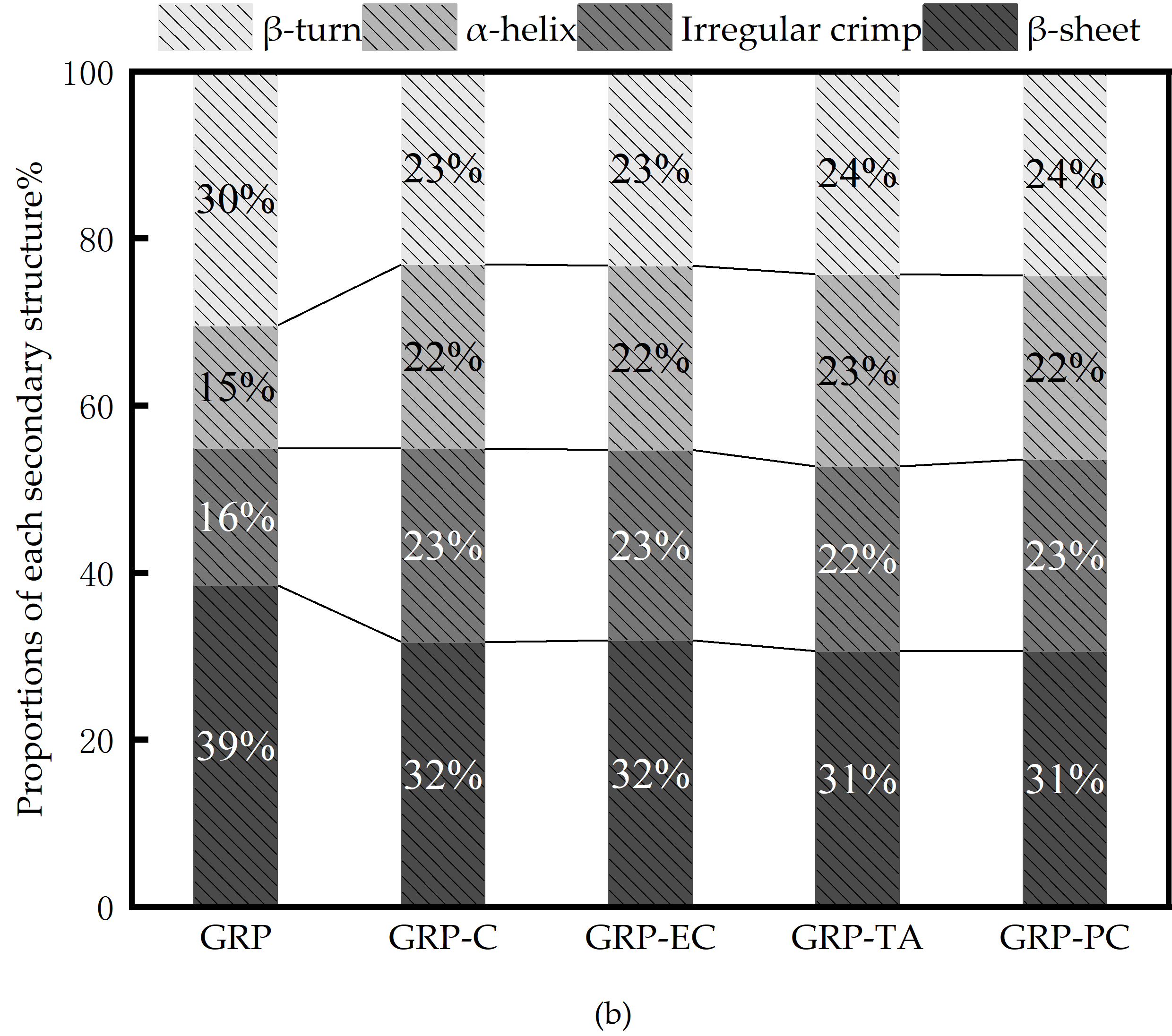

Supplement: Supplementary file 1 [file foods-15-01854-s001.zip › FigureS1-2.tif]
